# Machine learning on multiple epigenetic features reveals H3K27Ac as a driver of gene expression prediction across patients with glioblastoma

**DOI:** 10.1371/journal.pcbi.1012272

**Published:** 2025-08-07

**Authors:** Yusuke Suita, Hardy Bright, Yuan Pu, Merih Deniz Toruner, Jordan Idehen, Nikos Tapinos, Ritambhara Singh

**Affiliations:** 1 Laboratory of Cancer Epigenetics and Plasticity, Department of Neurosurgery, Brown University, Providence, Rhode Island, United States of America; 2 Data Science Institute, Brown University, Providence, Rhode Island, United States of America; 3 Center for Computational Molecular Biology, Brown University, Providence, Rhode Island, United States of America; 4 Department of Computer Science, Brown University, Providence, Rhode Island, United States of America; 5 Brown RNA Center, Brown University, Providence, Rhode Island, United States of America; National Institutes of Health, UNITED STATES OF AMERICA

## Abstract

Epigenetic mechanisms play a crucial role in driving transcript expression and shaping the phenotypic plasticity of glioblastoma stem cells (GSCs), contributing to tumor heterogeneity and therapeutic resistance. These mechanisms dynamically regulate the expression of key oncogenic and stemness-associated genes, enabling GSCs to adapt to environmental cues and evade targeted therapies. Importantly, epigenetic reprogramming allows GSCs to transition between cellular states, including therapy-resistant mesenchymal-like phenotypes, underscoring the need for epigenetic-targeting strategies to disrupt these adaptive processes. Understanding these epigenetic drivers of gene expression provides a foundation for novel therapeutic interventions aimed at eradicating GSCs and improving glioblastoma outcomes. Using machine learning (ML), we employ cross-patient prediction of transcript expression in GSCs by combining epigenetic features from various sources, including ATAC-seq, CTCF ChIP-seq, RNAPII ChIP-seq, H3K27Ac ChIP-seq, and RNA-seq. We investigate different ML and deep learning (DL) models for this task and ultimately build our final pipeline using XGBoost. The model trained on one patient generalizes to other 11 patients with high performance. Notably, H3K27Ac alone from a single patient is sufficient to predict gene expression in all 11 patients. Furthermore, the distribution of H3K27Ac peaks across the genomes of all patients is remarkably similar. These findings suggest that GSCs share a common distributional pattern of enhancer activity characterized by H3K27Ac, which can be utilized to predict gene expression in GSCs across patients. In summary, while GSCs are known for their transcriptomic and phenotypic heterogeneity, we propose that they share a common epigenetic pattern of enhancer activation that defines their underlying transcriptomic expression pattern. This pattern can predict gene expression across patient samples, providing valuable insights into the biology of GSCs.

## Author summary

This study aimed to develop a machine learning (ML) pipeline that can be used to investigate the role of epigenetic regulation on gene transcription in glioblastoma stem cells (GSCs). We developed a cross-patient prediction pipeline with multi-epigenomic data of patient-derived GSCs to predict gene expression. Our pipeline includes feature importance, which examines the impact of different epigenetic regulators, including chromatin accessibility (ATAC-seq), distal chromatin looping (CTCF ChIP-seq), histone modifications (H3K27Ac ChIP-seq), and active transcription (RNAPII ChIP-seq), on gene transcription across patients. Our analysis reveals that H3K27Ac is essential and sufficient to accurately predict gene expression across patients, suggesting that a common enhancer activation landscape defines gene expression in heterogenic patient-derived GSCs.

## 1. Introduction

Glioblastoma stem cells (GSCs) are characterized by tumor-initiating and self-renewal properties. They are central to the aggressive nature, therapy resistance, and recurrence of glioblastoma (GBM), the most lethal brain tumor [1–5]. These malignant properties and adaptability to tumor microenvironment are closely linked to GSC’s epigenetics. Understanding GSC’s epigenetic mechanisms not only provides new insights into GBM’s biology but also opens new avenues for therapeutic interventions.

Numerous epigenetic mechanisms and regulators influence gene transcription in physiology and disease. Amongst all epigenetic regulators, RNA Polymerase II activity, chromatin accessibility, CTCF-mediated genome organization, and H3K27 acetylation provide a comprehensive framework for understanding transcriptional regulation, as these mechanisms collectively govern gene expression dynamics. Together, these mechanisms integrate genetic and epigenetic cues, orchestrating transcriptional responses to developmental, environmental, and pathological signals. Investigating their interplay offers a holistic understanding of how gene expression is regulated, providing essential insights into normal physiology and disease states such as cancer.

RNA Polymerase II (RNA Pol II) is the central enzyme responsible for transcribing protein-coding genes and many non-coding RNAs, playing a crucial role in gene expression regulation. Its activity is tightly controlled through promoter recognition, transcription initiation, elongation, and termination. The phosphorylation state of its C-terminal domain (CTD) coordinates the recruitment of transcription factors, chromatin modifiers, and RNA processing machinery. RNA Pol II governs cellular responses to environmental cues, differentiation programs, and aberrant transcription in diseases like cancer.

Chromatin accessibility is a critical determinant of transcriptional activity, influencing the ability of transcription factors and RNA Pol II to engage with DNA. Open chromatin regions, often marked by nucleosome depletion and active histone modifications, allow for the recruitment of transcriptional machinery, while compacted heterochromatin restricts access and represses gene expression. Changes in chromatin accessibility play a fundamental role in cell fate decisions, stress responses, and oncogenic transformation, making it a key regulatory layer in gene expression control.

Histone H3 lysine 27 acetylation (H3K27Ac) is a key epigenetic modification associated with active transcription and enhancer activation. This acetylation mark, catalyzed by histone acetyltransferases (HATs) such as CBP/p300, distinguishes active enhancers from poised or inactive ones, facilitating the recruitment of transcriptional coactivators and RNA Polymerase II. By weakening histone-DNA interactions, H3K27Ac promotes chromatin accessibility, enabling transcription factors to engage with regulatory elements and drive gene expression. The presence of H3K27Ac at enhancers and promoters is critical for context-dependent gene activation in development, differentiation, and response to environmental signals. Its dysregulation is frequently observed in diseases like cancer, where aberrant enhancer activity leads to the misexpression of oncogenes and transcriptional reprogramming.

CCCTC-binding factor (CTCF) is a multifunctional DNA-binding protein that plays a pivotal role in genome organization by establishing chromatin loops, insulating regulatory elements, and facilitating higher-order chromatin architecture. By binding to specific DNA motifs, CTCF acts as a key mediator of topologically associating domains (TADs), influencing gene expression by either promoting or restricting enhancer-promoter interactions. It also functions as a transcriptional insulator, preventing the spread of repressive chromatin marks and maintaining genomic stability. Through these mechanisms, CTCF is essential for regulating cell-specific transcriptional programs, safeguarding genome integrity, and contributing to processes such as development, differentiation, and disease progression, including cancer [6–13].

The role of enhancer, RNAPII, chromatin accessibility, and CTCF is highly context-dependent, reflecting the intricate and interconnected nature of the epigenetic mechanisms. This complexity complicates the investigation of the relative contribution of these mechanisms on gene transcription regulation. Thus, integrative analysis is essential to resolve this and uncover the precise mechanisms that drive gene regulation in GSCs. Machine learning (ML)-based gene expression prediction has been used to extract patterns from the epigenomics big data such as Assay for Transposase-Accessible Chromatin using sequencing (ATAC-seq), Chromatin Immunoprecipitation sequencing (ChIP-seq) of histone modification marks or transcription factors and to distinguish the contribution of multiple epigenetic markers [14–20]. A convolutional neural network-based model built with two types of epigenetic modulators (chromatin accessibility and histone modifications) performed better than one built with a single epigenetic modulator [14]. ML-based models, including XGBoost with genomic data, histone modifications, and chromatin looping, were able to predict gene expression and were used to interpret the contributions of those features [21]. GC-MERGE takes histone modifications, including H3K27Ac, an enhancer marker, and chromatin looping, defined by Hi-C, using graph convolutional networks to predict gene expression [22]. GraphReg takes histone modifications, including H3K27Ac and chromatin accessibility data, defined by DNase-seq, using convolutional layers, and Hi-C data, using graph attention networks, to predict gene expression [23]. These studies highlight the importance of using multiple types of epigenetic markers to predict gene expression. However, these models were developed using cell lines or primary cells from a single source and cannot be applied to cells across patient datasets, thus providing limited value in understanding the biology of human glioblastoma.

Cross-patient prediction is a critical method to advance the understanding of complex epigenetic mechanisms and correlate the findings to human disease [24]. When a cross-patient prediction model is generalized across datasets, this model is scalable and can be used as a tool across various populations or research settings without requiring extensive fine-tuning. We develop an ML-based cross-patient prediction model - **C**ross patient-**I**nformed **P**rediction of **H**uman **E**pigenetic **R**egulation (CIPHER) - that predicts gene expression across patient-derived GSCs. We benchmark several ML and Deep Learning (DL) models on our GSC patient-derived dataset with multiple epigenetic regulators (ATAC-seq, RNAPII ChIP-seq, CTCF ChIP-seq, and H3K27Ac ChIP-seq) as input and find that XGBoost gives the best empirical performance. Next, we investigate the relative contribution of each epigenetic feature to gene expression prediction by feature analysis. This analysis reveals that H3K27Ac, the chromatin mark that defines active enhancers, is sufficient to predict gene expression across our two patient samples. Finally, we apply our trained CIPHER model to 10 additional publicly available GSC patient samples with H3K27Ac ChIP-seq/RNA-seq, showing that CIPHER generalizes to data across patients. Furthermore, we trained CIPHER on H3K27Ac ChIP-seq alone and found that it gave better predictions than the model trained with all the epigenetic features. The investigation of the H3K27Ac signal patterns across patients shows a similarity between high- and low-expressed genes, which aligns with the cross-patient model performance. Therefore, our results support our hypothesis that the landscape of enhancer activity can act as a “blueprint” for transcriptional regulation in GSCs, and they imply a common chromatin pattern around active enhancers in GSCs that dictates gene expression.

## 2. Materials and methods

### 2.1 Datasets and pre-processing

**Fig 1:**
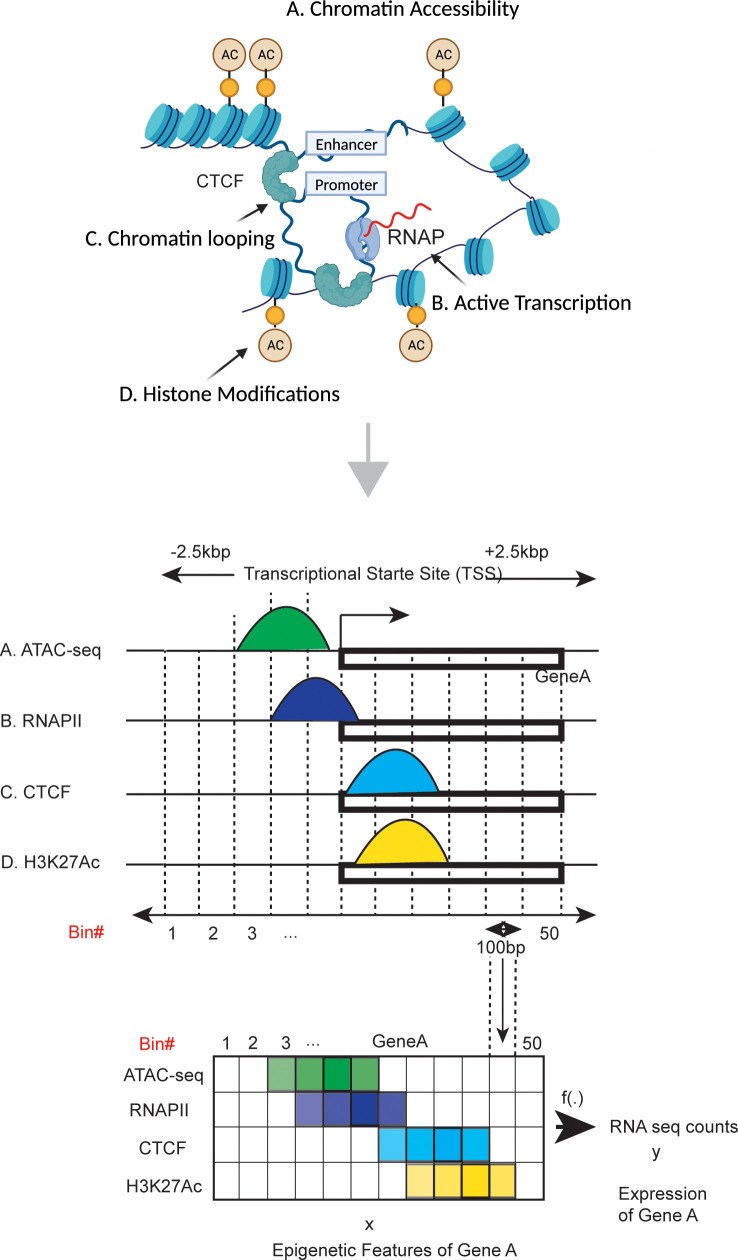
Schematic overview of epigenetics-driven gene transcription and epigenomics sequencing data processing. Gene transcription on epigenetic mechanisms that can be categorized into four categories: A) Chromatin accessibility, B) Active Transcription, C) Chromatin looping, D) Histone modifications. Counts of these sequencing + /- 2.5 kilo base-pairs (kbp) flanking the TSS region of each gene were measured and divided into 50 bins, with each bin representing 100 base pairs to create a heatmap for the input of the model. Created with BioRender.com.

We model and investigate the relationship between epigenetic modulators and gene transcription of patient derived GSCs using CIPHER. To achieve this, we used the following four markers to compose our GSC patient datasets: ChIP-sequencing with H3K27Ac (enhancer marker), RNAPII (active transcription marker), and CTCF (distal chromatin looping marker), and ATAC-sequencing (chromatin accessibility) and RNA-sequencing data (Fig 1). This dataset was created for two patient-derived GSCs collected in our lab (GSC1 and GSC2). For testing model generalizability, we additionally included 10 datasets composed of the H3K27Ac marker and RNA-seq data, from the Mack et al. study [25]. We select the 10 sets of data randomly from the 44 available sets of GSC measurements. For detailed information on the preparation of these datasets, see Section S1 in S1 Text.

As ML modeling for GSC patient derived samples has not been explored before, we first perform a correlation analysis between gene expression and each epigenetic modulator (ATAC-seq, H3K27Ac ChIP-seq, CTCF ChIP-seq, and RNAPII ChIP-seq) for our two GSC samples. We observe a positive correlation of 0.366, 0.111, 0.208, and 0.333 (for H3K27Ac, CTCF, ATAC-seq, and RNAPII, respectively) in GSC1, and 0.412, 0.162, 0.221, and 0.359 (for the same order of modulators) in GSC2 (S4A and S4B Fig). Therefore, we hypothesize that ML models can learn the relationship between epigenetic modulators and gene expression.

**Fig 2:**
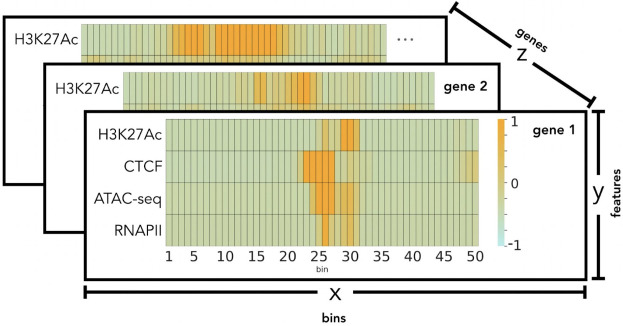
Patient datasets representation after preprocessing. The figure depicts the standardized epigenetic marker values per gene for a patient. It highlights the 3-dimensional arrangement of the datasets prior to model input. Here the X axis corresponds to the 50 bins of 100 bp counts for each feature. The Y axis represents each gene’s 4 epigenetic features. The figure’s Z axis is representative of the gene arrangement in the dataset.

We focus on the + /- 2.5 kilo base-pairs (kbp) flanking region of the transcription start site (TSS) for each gene and divide it into 50 bins, with each bin representing 100 base pairs - this bin resolution was determined based on previous studies [22,26]. To prepare the genomic data for ML modeling, we create a 50 x 4 matrix with rows representing bins and columns for epigenetic features for each of the 20,015 genes, and each bin contains summarized counts. As a result, we pass 20,015 x 50 x 4 (1,000,750 samples per feature) to our model as an input. Therefore, each gene is a data sample to train the model, with its 50x4 matrix representing the 4 epigenetic signals as input vectors of size 50 and its expression as the output. To prepare the gene expression output labels, we summarize counts of the + /- 2.5kb flanking the TSS per gene and normalize it using transcripts per million (Fig 2).

The second preprocessing step includes separate standardization and log(2) transformation of the gene expression measurements [19]. To account for the variation in count values across different types of sequencing and experimental conditions, we standardize the counts of each sequencing data by using the mean value of the corresponding sequence data. This standardization occurs after the training, validation, and test data-splitting is performed. Here, each of the four epigenetic features is standardized separately within the training, validation, and test sets. We choose to standardize the data at this point, as opposed to before the data splitting, to avoid potential data leakage [27]. Each gene has its corresponding target label log(2) transformed with a pseudo count of 1, prior to the data split process. Additional information regarding the model input process is contained in Section S2 in S1 Text and S1 and S2 Figs.

### 2.2 Machine learning modeling

We test several regression-based ML and DL models to examine the novel application of our specific combination of epigenetic markers to predict gene expression in a cross-patient setting for CIPHER. Given the two patients (GSC1 and GSC2), we seek to use ML to extract a common pattern seen across patients, as GSCs are highly heterogeneous from one patient to the next. Therefore, for hyperparameter tuning and preliminary validation, we train each model by splitting GSC1’s dataset into 70% for training and 30% for validation (non-overlapping gene sets) in a within-patient cross-chromosomal setting [28]. For each model, we perform hyperparameter tuning using a grid search optimized for Pearson Correlation Coefficient (PCC). Section S3 in S1 Text and S1-S8 Tables includes additional information on hyperparameter tuning. Once we have selected the final set of hyperparameters, we train the ML model with the full set (100%) of gene observations from GSC1 (patient 1) and test it for GSC2 gene expression prediction for all the genes (represented as GSC1 → GSC2). The results from the cross-patient setting are reported in the Results Section (Section 4 in S1 Text).

In every case, the prediction task is a regression where the models predict the RNA-seq gene expression value per gene. Cross-patient prediction experiments require two datasets (one for training and validation, and the other for testing), each composed of ChIP-seq, ATAC-seq, and RNA-seq.

To select the best machine learning model, we comprehensively test deep learning architectures like a Multi-layered Perceptron (MLP), a Convolutional Neural Network (CNN), a Recurrent Neural Network (RNN), and a Branched Multi-layer Perceptron (Branched MLP). With the branched MLP, we combined the genomic sequences from the region as inputs.

Traditional machine learning algorithms include Gradient Boosting Regression (GBR), Support Vector Regression (SVR), and Multiple Linear Regression (MLR) architectures. Detailed information regarding these models is outlined in Section S3 in S1 Text.

Additionally, a traditional data splitting framework where the training and testing data is from the same distribution and therefore the model is tested on held out genes (within-patient cross-chromosomal) is evaluated to support our cross-patient experiments. Further details and results are in Section S9 in S1 Text.

**Evaluation metrics.** Pearson Correlation Coefficient (PCC) is used as the primary metric for hyperparameter tuning, performance evaluation, and feature perturbation. We include Spearman Correlation Coefficient (SCC) as an additional evaluation metric.

**Computational considerations.** Script time considerations are detailed in the Section S6 in S1 Text and S9 Table.

#### 2.2.1 Criteria for model selection.

Out of all the models tested, we select our final machine learning model based on the analysis of each model’s overall Pearson Correlation Coefficient (PCC) score results.

#### 2.2.2 XGBoost model details.

**Fig 3:**
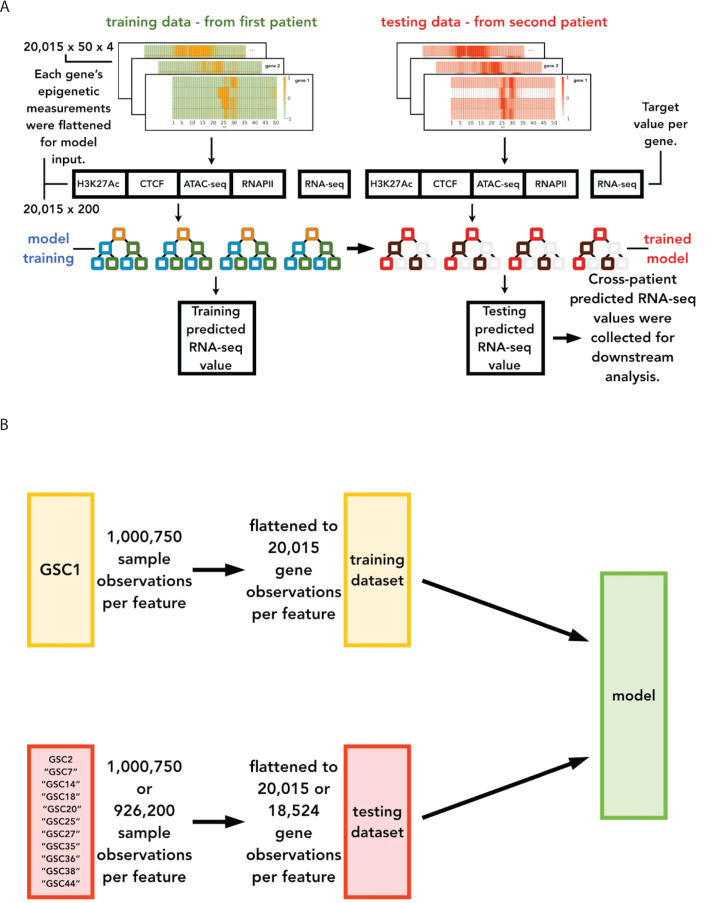
Cross-patient prediction methodology using the model XGBoost architecture. The model input for training and validation is derived from a patient (GSC1) different from the testing dataset. As shown, the matrices are flattened before going into the model, where the RNA-seq value is predicted. A) A functional view of the cross-patient experimental setup where the model training is illustrated on the left side of the image and the right, the transition to testing with the trained model. B) A conceptual view of the cross-patient experimental setup, which illustrates the dataset allocation and number of observations per feature in the data for training and testing.

The XGBoost based model exhibits the best average performance of all the cross-patient experiments in this study. Since XGBoost takes 2-dimensional input, the dataset features are flattened to 20,015 x 200 while the target variable (RNA-seq) becomes a 20,015 x 1 array. The relative positioning of the bins of each of the four gene features is kept contiguous (Fig 3A). Therefore, we choose this formulation with XGBoost as our CIPHER model for all subsequent testing and analysis.

**Loss function.** We use mean squared error (MSE) for loss function calculations as follows:


$$mean\;squared\;error\;=\frac{1}{n}\sum_{i=1}^{n}(y_{i\;}-\hat{y_{i}}{)}^{2}$$ (1)

In the calculation $y_{i\;}$ represents the actual RNA-seq measurement while $\hat{y_{i}}$ represents the predicted value per gene i for the number of genes n in the dataset.

### 2.3 Model testing across multiple seeds

We ran each model 10 times (with different random seeds) in the cross-patient arrangement (GSC1 → GSC2) to evaluate the robustness of the results. The mean, standard deviation, and distribution visualization of our metrics are reported for our study’s experimental results.

When we apply CIPHER to the additional datasets from Mack et al. (S1), the trained model is evaluated with the entire test GSC dataset (100%) (Fig 3B) [25]. Each evaluation dataset was run 10 times with a different random seed.

### 2.4 Model testing across subsets of testing datasets

For completeness, we also investigate the variance in the model performance across subsets of each testing dataset. To accomplish this, the training dataset (GSC1), the model training process, and the model hyperparameters remain the same. Instead of evaluating the trained model with all the samples in each testing dataset (GSC2 and all of the Mack et al. derived datasets) at once, each of the testing datasets are split into 10 parts [25]. The gene information present in each part does not overlap with any other part. This strategy, as opposed to some form of random selection for the subsets, ensures that every gene’s values are evaluated and not to inadvertently exclude any potential genes of interest. For GSC2 parts 1–9 include 2,002 genes each. GSC2 part 10 includes 1,997 genes (99,850 samples per epigenetic feature). Meanwhile, each Mack-GSC parts 1–9 include 1,853 genes each (92,650 samples per epigenetic feature) and their 10th subset includes 1,847 (92,350 samples per feature).

The trained model is evaluated with each testing subset together with a different random seed. The means and standard deviations of these additional results (S10) follow the same trends for all the results discussed below.

### 2.5 Perturbation experimental setup and analysis

To investigate the effect each individual epigenetic signal has on prediction, we apply a method which alters one of the four epigenetic features of the GSC2 dataset, at a time. Specifically, perturbation of a particular epigenetic signal was achieved by replacing all of that feature’s values with 0.0 (the mean of the standardization) for all gene’s across the test dataset. The trained model is then evaluated with the altered dataset 10 times, with a different random seed each time. The analysis compared the calculated mean and standard deviation results for each set of experiments, to the original model performance and each other (S7).

## 3. Results

### 3.1 Machine and deep learning models perform similarly in different cross-patient prediction scenarios

**Fig 4:**
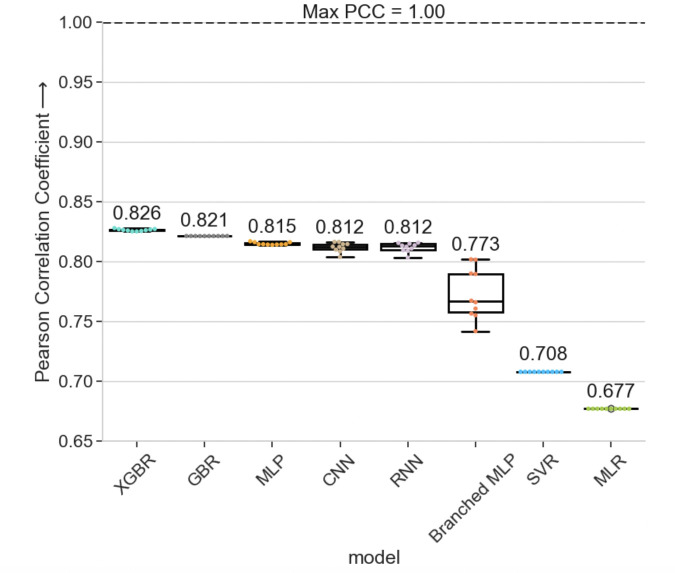
PCC cross-patient regression model results. Our experimental results are compiled as the mean PCC scores over 10 runs of each model. The error bars shown indicate standard deviation of the model results. Our cross-patient XGBoost-based regression model performed higher than all other architectures when training with GSC1 and testing with GSC2 (GSC1 → GSC2).

We find that when the models trained with GSC1’s (patient 1) and tested with GSC2’s (patient 2) data, which we refer to as GSC1 → GSC2, XGBoost Regression (XGBR) model achieves the highest PCC scores with the of 0.826199 ± 0.000888. Multiple Linear Regression (MLR), on the other hand, gives the lowest performance of 0.676872 ± 0.0; a 0.149327 difference in PCC (Fig 4). The sub-par performance of MLR indicates that the relationships among the epigenetic markers and their relationship with gene expression are non-linear. The remaining models perform within 0.014497 of each other, suggesting that they, too can capture the non-linearity in the data effectively. This indicates that the results are, to some degree, modestly affected by the model used and more so dependent on the epigenetic features of the dataset.

The high PCC scores highlight the success of our cross-patient prediction approach to generalize the epigenetic input patterns connected to the gene expression from one patient to another. Note that the XGBR PCC scores are considerably higher than the simple correlation analysis we performed before and the linear regression results. This result suggests that the finer resolution input of the epigenetic modulators into the XGBR model allows it to better learn the non-linear relationships between them and the gene expression. Our study’s corresponding SCC score results also present the XGBR model with the highest SCC score. The GBR and MLP exchange places but are behind XGBR. Meanwhile, the MLR model is again the least performant, with the lowest mean SCC score. Our SCC results are detailed in the Section S5 in S1 Text and S5 Fig. We select XGBR as our final CIPHER model architecture based on the overall better empirical performance.

### 3.2. Active enhancer, defined by H3K27Ac epigenetic state, by itself is sufficient to predict gene expressions in GSCs across patients

To interpret the importance of each epigenetic marker in predicting gene expressions, we calculate the feature importance for the XGBoost architecture of CIPHER. Since the datasets were flattened into two-dimensional inputs, the importance scores are calculated at the bin (or genomic region) level. Therefore, we sum the individual feature importance scores corresponding to the four epigenetic markers.

**Fig 5:**
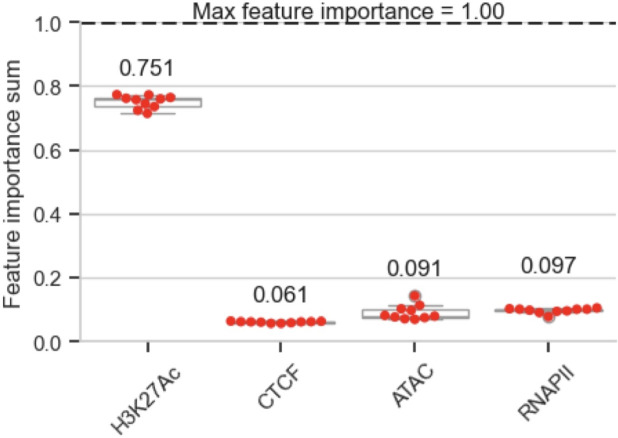
Feature importance scores extracted from our cross-patient CIPHER model for GSC1 → GSC2. The model identifies the H3K27Ac feature as the most important for the prediction of RNA-seq. The results visualized are the means over 10 experimental runs, with the error bars denoting the standard deviation.

All the combined epigenetic markers (H3K27Ac, RNAPII, ATAC-seq, and CTCF) contribute to gene expression prediction to a variable extent (Fig 5). As a complementary analysis, we also perform perturbation analysis, where we remove one epigenetic factor and observe a decline in prediction performance to assess the contribution of epigenetic markers. Each epigenetic marker perturbation leads to a decline in PCC, suggesting each epigenetic marker contributes to predicting gene expression (S7, S9 and S10 Figs).

Interestingly, the H3K27Ac marker is the most important feature for RNA-seq prediction by a considerable margin. The maximum possible importance value is 1.0, and the summed values for H3K27Ac amounts to 0.750977 ± 0.020292. This is followed by RNAPII (0.096962 ± 0.007921), ATAC-seq (0.091166 ± 0.023353), and CTCF (0.060894 ± 0.002569) (Fig 5). The order of importance (H3K27Ac, followed by RNAPII, ATAC-seq, CTCF) aligns with the order of the correlation between each epigenetic marker and RNA-seq (S4, S4A, and S4B Fig). This H3K27Ac marker’s considerable importance, followed by small feature importance values of RNAPII, ATAC-seq, and CTCF, is potentially due to the overlapping activity of these markers on gene expression. This is supported by the positive correlation between the H3K27Ac signal, enhancer marker, and RNAPII, ATAC-seq, and CTCF signals (S4, S4A and S4B Fig).

When we consider the strengths of our feature importance observations, we consider that each marker’s measurements make up 25% of the GSC1 and GSC2 patient datasets. Since each of the four markers is equally represented in both the GSC1 training dataset and the GSC2 dataset used in the evaluation, the observation that the feature importance calculated by the model speaks to our comments on the relative importance of each marker for prediction. The notion that PCC is affected by the perturbation of each signal to varying degrees and our discussion of H3K27Ac’s importance is supported by this equal representation as well (S7).

### 3.3 Cross-patient analysis with additional patient GSC H3K27Ac epigenetic data demonstrates CIPHER’s cross-patient generalization ability

**Fig 6:**
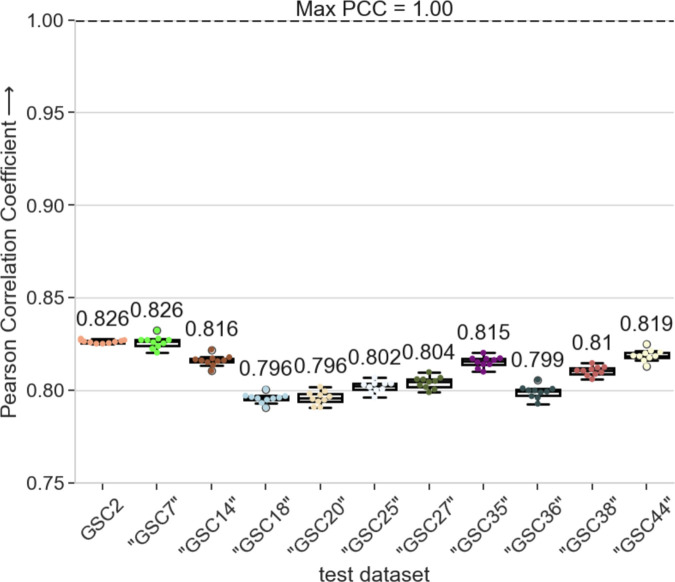
CIPHER Model training generalizes to other GSC datasets. The CIPHER model trained with the GSC1 dataset is then evaluated with the GSC H3K27Ac/RNA-seq data from Mack et al [25]. In line with the other study experiments, each dataset is evaluated 10 times, with 10 different seeds. These experiments uncovered the similarity between the GSC data from different sources.

To validate our cross-patient approach and investigate H3K27Ac as the main contributor to predicting gene expression in a larger population of patients, we apply our trained CIPHER model to the only other publicly available GSC’s H3K27Ac and RNA-seq data from Mack et al. study [25]. We input the GSC H3K27Ac signal to our trained CIPHER model. Since this dataset does not have other epigenetic signals, we use zeros as inputs for the other features and measure the performance using PCC. Note we do not perform any re-training or fine-tuning of CIPHER using this dataset. The PCC scores of all the additional 10 sets are similar to that of our initial sample (GSC2), suggesting that our cross-patient approach is generalizable to the GSC data from independent studies (Fig 6). Along with the prominent contribution of the H3K27Ac, active enhancer marker, on predicting gene expressions, we show that, across patient populations, H3K27Ac emerges as a higher predictive signal compared to RNAPII, ATAC-seq, and CTCF.

**Fig 7:**
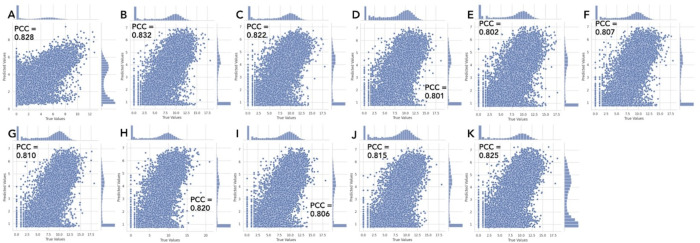
RNA-seq true versus predicted value scatter plots illustrate a similar predictive trend across GSC datasets. A) GSC2, B) Mack-GSC7, C) Mack-GSC14, D) Mack-GSC18, E) Mack-GSC20, F) Mack-GSC25, G) Mack-GSC27, H) Mack-GSC35, I) Mack-GSC36, J) Mack-GSC38, K) Mack-GSC44.

To observe the general prediction trends, we plot the true and predicted values of RNA-seq of GSC2 and all the additional GSCs from the other study. We observe that the true RNA-seq values of GSC2 range from 0 to 12.762008. Meanwhile, the other testing GSCs range from 0 to 17.674399 at their minimum, with a maximum of 21.003699. Additionally, the training set GSC1, has a range of 0 to 12.762008. Despite the difference in these ranges, the predicted RNA-seq values across all the testing GSCs produce plots that share similar trends (Fig 7A-K).

The CIPHER model is trained using all the epigenetic features of the GSC1 dataset. Each plot is representative of the model run (out of the 10 runs per dataset) that produced the highest PCC for that dataset. The axes represent RNA-seq count values after the log2 transformation. The true data values of GSC2 (panel A) have a different range (0 to 12.762008) than the others (0 to 21.003699), as shown on the X-axes. The predicted values (Y-axes) have more similar ranges due to the model being trained with the target RNA-seq of GSC1, which is up to 12.652625.

### 3.4 Cross-patient model experimentation isolated to training/testing with GSC’s H3K27Ac data highlights its influence on predicting gene expressions

To directly evaluate how much predictive power is retained with H3K27Ac alone and how its contribution compares to the combined impact of all the epigenetic makers, we re-train CIPHER using only the H3K27Ac feature and omit the RNAPII, ATAC-seq, and CTCF features while keeping the original set of hyperparameters. We perform this analysis using the same setup of GSC1 sample as the training data and GSC2 and the additional GSCs as testing data to ensure consistency. To quantify the model’s performance, we measure the mean PCC and compare it to the PCC obtained when all the features (H3K27Ac, RNAPII, ATAC-seq, and CTCF) are included.

Interestingly, the PCC score of CIPHER with only the H3K27Ac feature is generally higher than that with all the features. In nine out of ten GSCs, the PCC with only the H3K27Ac feature is higher than that with all the features by 0.994-2.62%. In one GSC (GSC2), the PCC with only the H3K27Ac feature is lower than that with all the features by 1.23% (Table 1). This discrepancy aligns with the fact that GSC2 is the only dataset with all the epigenetic markers (H3K27Ac, RNAPII, ATAC, and CTCF), whereas the other GSCs had only H3K27Ac available. This highlights the sufficiency of active enhancer signals (marked by H3K27Ac) in predicting gene expression across patients, even from different distributions, strongly suggesting the critical role of enhancer activity in gene transcription regulation.

**Table 1:** Training and testing with H3K27Ac only resulted in an increase in mean PCC for most of the datasets.

dataset	all features	H3K27Ac only	difference	percent change
**GSC2**	0.826199±0.000888	0.816033±0.000321	-0.010166	-1.230454%
**Mack-GSC7**	0.825879±0.003283	0.841503±0.000591	0.015624	1.891803%
**Mack-GSC14**	0.816024±0.002940	0.82373±0.000629	0.007706	0.944335%
**Mack-GSC18**	0.795605±0.00259	0.811216±0.000501	0.015611	1.962155%
**Mack-GSC20**	0.795859±0.003535	0.812941±0.000621	0.017082	2.146360%
**Mack-GSC25**	0.801711±0.003055	0.822755±0.000673	0.021044	2.624886%
**Mack-GSC27**	0.804101±0.003224	0.824224±0.000755	0.020123	2.502546%
**Mack-GSC35**	0.815198±0.002965	0.835197±0.000804	0.019999	2.453269%
**Mack-GSC36**	0.799017±0.003429	0.818522±0.000625	0.019505	2.441125%
**Mack-GSC38**	0.810365±0.002465	0.828615±0.000501	0.01825	2.252072%
**Mack-GSC44**	0.818837±0.003144	0.834236±0.000824	0.015399	1.880594%

This table compiles the PCC scores of CIPHER model trained with all the epigenetic markers (“all features”) and CIPHER model trained with only H3K27Ac (“H3K27Ac only”), the difference between those two with the base on “all features” (“difference”), and the percent change from “all features” to “H3K27Ac only” (“percent change”). While the PCC decreased with GSC2, all the other datasets resulted in an increase of roughly 0.9-2.6%.

**Fig 8:**
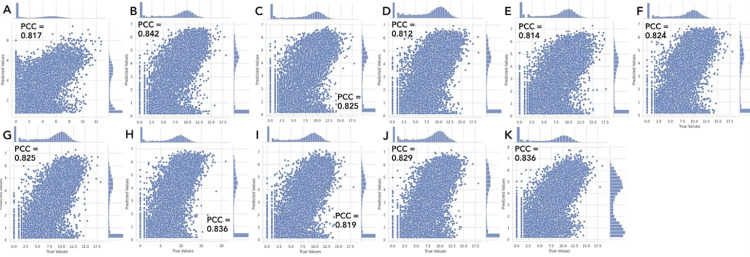
H3K27Ac only model produced RNA-seq true versus predicted value scatter plots like the prior experimental setup. A) GSC2, B) Mack-GSC7, C) Mack-GSC14, D) Mack-GSC18, E) Mack-GSC20, F) Mack-GSC25, G) Mack-GSC27, H) Mack-GSC35, I) Mack-GSC36, J) Mack-GSC38, K) Mack-GSC44. Each plot is representative of the model run (out of the 10 runs per dataset) that produced the highest PCC for that dataset and the axes represent RNA-seq count values after log2 transformation. The ranges of both the true and predicted values for each dataset follow the previous testing closely. Additionally, as in the previous testing, the predicted values histograms’ shapes and sizes in each visualization differ from each other but follow the same trends.

To observe the prediction trends, we plot the true and predicted values of RNA-seq of GSC2 and all the additional GSCs from the other study again and compare to the previous plots with all the epigenetic features. These scatter plots of the true and predicted values of RNA-seq with only H3K27Ac are similar to the previous plots with all the epigenetic features (Figs 7 and 8). This indicates the cross-patient gene expression prediction relies on only H3K27Ac, not the other epigenetic features, suggesting that an active enhancer is sufficient to predict gene expressions across patients.

### 3.5. CIPHER learns the common pattern of H3K27Ac to predict gene expression in GSCs despite their known heterogeneity

**Fig 9:**
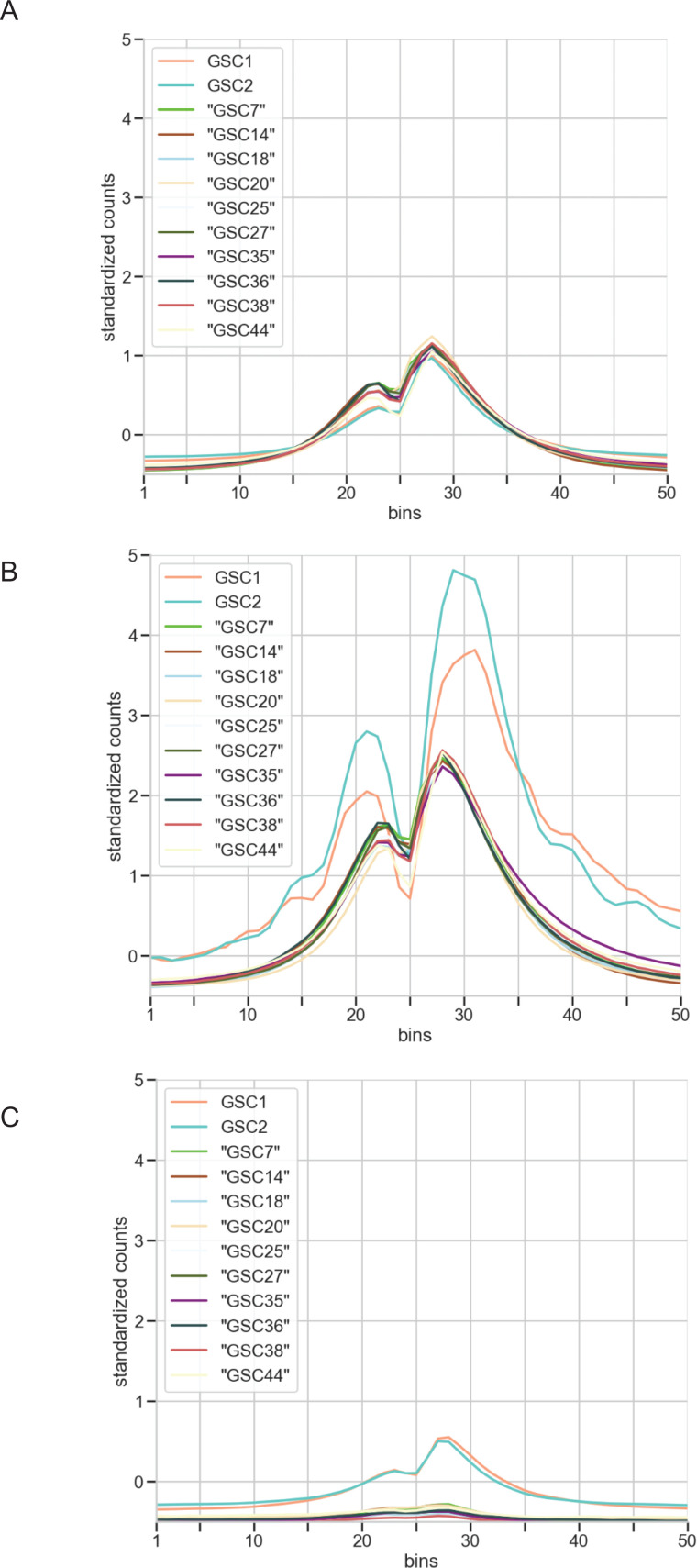
An analysis of H3K27Ac standardized counts visualizes the similarities in signal shape contrasted by the variations at peaks. H3K27Ac counts of all the GSC datasets around TSS are visualized at bin levels. A) Visualizing H3K27Ac signals for all genes, regardless of their RNA-seq values. All these GSCs have similar H3K27Ac epigenetic landscapes with two distinct peaks B) H3K27Ac signals for “high” expressing genes (log2(RNA-seq values) ≥ 10) C) H3K27Ac signals for “low” expressing genes (0<log2(RNA-seq values) ≤5).

To further interpret the bin/genomic-level contribution of H3K27Ac signals around TSS regions on predicting gene expression, we visualize the means of all the gene’s H3K27Ac counts around TSS regions within each GSC dataset. All the GSCs exhibit the same H3K27ac distribution with uniform magnitudes despite coming from different studies (Fig 9A). This indicates the model’s consistent ability to learn H3K27Ac patterns and to predict the appropriate gene expression across the datasets. Moreover, it suggests that the cross-patient prediction setting doesn’t necessarily constitute a major distribution shift. To further investigate the relationship between these H3K27Ac signals and the model’s ability to generalize, we visualize the subset of genes that have relatively “high” true expression values (log2(RNA-seq values) ≥10). We observe that both GSC1 and GSC2 have more pronounced peaks and associated declines at bin 25 than the other GSCs (Fig 9B). Given that the model is trained with GSC1, which contains a lower number of “high” expression genes (68), along with a lower maximum expression value (12.652625) in the dataset, the model does not have the same effectiveness predicting the “high” expression genes of the other GSCs. Conversely, when we visualize the pattern for genes with “low” true expression values (0<log2(RNA-seq values) ≤5) we observe that the H3K27Ac signals are lower across the bins for all the GSC data compared to the “high” expression and the overall means (Fig 9C). A higher number of genes in “low” expression compared to “high” expression within the GSC1 data indicates the positive impact that the “low” category has on overall PCC and the emergent model generalization we note across all the GSCs. Section S8 in S1 Text includes examples of gene expression category population numbers and associated model errors.

Overall, the presence of the H3K27Ac peaks in the “high” expression group and the absence of those in the “low” expression group indicates that our cross–patient model learns the common pattern of H3K27Ac around TSS regions to predict gene expression levels. This suggests that GSCs share a common pattern of active enhancer marks that determines transcriptional expression levels despite the known heterogeneity of GSCs.

## 4. Discussion

The origin and maintenance of GSC adaptability are regulated by intrinsic cell processes affecting DNA, chromatin, and RNA, as well as by external microenvironment factors that help propagate cancer stem cell phenotypes. Epigenetic mechanisms play an important role in maintaining GSCs and supporting tumor persistence [29–31].

To model the non-linearity of epigenetic mechanisms that drive gene expression, particularly the relative contribution of multiple types of epigenetic markers, we built CIPHER, a machine learning framework based on a combination of epigenetic bulk NGS data. Our goal was to discover epigenetic marks that can predict gene expression across patients with GBM and to provide data-driven systematic evaluation of multiple epigenetic features. A comprehensive evaluation of different ML models demonstrates that XGBR architecture is the best-performing model (PCC = 0.826), which is better than traditional correlation analysis (0.366 - 0.412) (Figs 3 and S4B). And this result is consistent with XGBR’s strength in processing tabular data [32]. Our datasets consisted of various types of epigenetic modulators structured as a series of tables. This aligns with a previous study that utilized XGBR, which incorporated genomic data, histone modifications, and chromatin looping and achieved high AUC (0.79-0.88) [21].

Several factors contribute to the high performance of our model: a) the particular set of epigenetic markers and b) how the input data was prepared. Previous studies identified H2Az and H3K4me3 as key contributors to the prediction of gene transcription, whereas our study highlights the use of H3K27Ac [21]. This reinforces the importance of active enhancers in predicting gene expression. In addition, previous studies have shown that signal contributions around promoter and gene body differ, whereas, in our model, the inclusion of +/- 2.5kb flanking TSS sites might have enhanced prediction performance.

Another significant finding of our study is the CIPHER’s generalizability across patients. Without any model re-training, CIPHER maintained high performance when applied to 11 GSCs patient dataset from independent studies without overfitting, indicated by the PCC (0.796-0.826) across GSCs (Fig 3). This performance is in line with previous reported PCC scores and better in some cases. Previous models such as GC-MERGE, despite the use of Graph Convolutional Model and Hi-C data within a single cell line, returned PCC (0.76-0.79) [22]. Similarly, GraphReg, which leveraged the CNN on histone modifications (including H3K27Ac) and chromatin accessibility and graph attention networks on H3K27Ac Hi-ChIP and Hi-C across cell lines, reported R² (0.578-0.607) [22]. The higher performance of CIPHER might be due to the advantage of XGBoost over CNN for histone modifier ChIP-seq, as seen in our model comparison (Fig 3). We also observe cross-patient generalization (Fig 6) performs better than within-patient, unseen gene generalization (S12A and S12B Fig). This might be due to the limited variation across patients or the redundancy in epigenetic patterns across genes. This suggests that gene-level variation poses a stronger challenge to the model than patient identity in this dataset.

Additionally, the differences in datasets might play a role on the CIPHER’s generalizability - GraphReg attempted to generalize from GM12878 (lymphoblastoid cell) to K562 (bone marrow lymphoblast cells) or vice versa, whereas our samples focus on GSCs from different patient-derived datasets. Regardless of the underlying contributors, the generalizability of CIPHER highlights its potential application to broader patient populations. More importantly, CIPHER provides a principled and interpretable framework for integrating multiple epigenetic features while enabling feature contribution analysis across different prediction settings and can identify epigenetic patterns beyond dataset-specific correlation analysis.

Our cross-patient feature importance analysis confirms the central regulatory role of H3K27ac, particularly between the chosen multiple epigenetic features. Combined with H3K27Ac epigenetic landscape analysis, it highlights that the landscape of active enhancers alone is sufficient to predict gene transcription in GSCs. However, we acknowledge that this XGBR-based model doesn’t capture some genes like those highly variable or expressed genes. These might require additional regulatory context that is not captured by our current epigenetic markers. Our perturbation analysis shows that the sum of the drop in PCC by each epigenetic modulator is less than PCC without perturbation (S7, S9 and S10 Figs). This indicates the existence of other potential contributors to predicting gene expression, such as other histone modification marks (e.g., H3K9me3, H3K27me3, etc.) and modulators of 3D chromatin structure.

The potential role of the active enhancer pattern in predicting gene expression in GSCs from different distributions of patients raises important questions and critical implications for translational applications. CRISPR-based enhancer perturbation studies have already demonstrated the potential of identifying enhancers critical for cancer cell proliferation [33–35]. Such enhancer patterns can serve as valuable potential biomarkers for diagnosis or identification of therapeutic targets. In the future, it will be important to identify targetable common epigenetic features around the active enhancers of GSCs with more confidence and to identify those. For future work, we will incorporate Hi-C data using a graphical convolutional network as seen in GC-MERGE and incorporate other DNA sequence data as seen in GraphReg by using a transformer. This approach of target identification can lead to design of epigeneti silencers that could inhibit oncogenic transcriptional programs in glioblastoma.

## 5. Conclusion

We build Cross patient-Informed Prediction of Human Epigenetic Regulation (CIPHER), a cross-patient prediction analysis framework that systematically evaluates the contribution of multi-epigenetics markers on predicting gene expression of cancer stem cells from different studies. By applying this framework to GSCs, we identify that H3K27Ac signals contribute most to predicting gene expressions, followed by RNAPII, ATAC-seq, and CTCF, and this contribution is preserved across patients. Furthermore, we input only H3K27Ac ChIP-seq to our trained model and show excellent performance. Our H3K27Ac landscape analysis shows a common landscape across GSCs from different studies, suggesting that the enhancer landscape is sufficient in predicting gene expression of GSCs across patients. Overall, we present CIPHER, a cross-patient gene expression prediction framework that can formulate deep insights into epigenetic-driven gene expression mechanisms and the epigenetic landscape of cancer stem cells.

## Supporting information

S1 Text **Section S1.** Epigenetic marker and RNA-sequencing information. **Section S2.** Flowcharts of data preparation and preprocessing process. **Section S3**. Supporting predictive models’ information [36–38]. **Section S4**. Correlation highlights the variation among the epigenetic features for each dataset. **Section S5.** Supporting model results. **Section S6.** Computational considerations. **Section S7.** Perturbation results support model feature importances. **Section S8.** Supporting gene expression count and model error analysis. **Section S9.** Single-patient experimental setup demonstrates comparable model prediction performance to cross-patient modeling and echoes the importance of H3K27Ac. **Section S10.** Model evaluation over subsets of data supports the trends uncovered in the results.
(DOCX)

S1 Fig Illustration of dataset preparation process. The process that extracts the observed epigenetic marker values from BAM format files uses a series of utilities which combine the separate data into one file. The process aligns the 50 bin values of the gene’s epigenetic features with a single extracted RNA-seq value for the gene to maintain consistent formatting.
(TIFF)

S2 Fig Illustration of data preprocessing process. The epigenetic feature values per gene are extracted, formed into matrices, and standardized for model input. Each gene’s observed RNA-seq value is extracted and transformed creating the target variable for the model.
(TIFF)

S3 Fig Illustration of the dataset processing process for the Mack et al. derived H3K27Ac and RNA-seq information [25]. We use this process to convert and adapt the downloaded H3K27Ac and RNA-seq information to align with our study’s existing preprocessing.
(TIFF)

S4 Fig Correlation within GSC1 and GSC2 datasets. The two visualizations convey the calculated correlation values for our two patient datasets. GSC1 is shown in panel **A** with GSC2 in panel **B**. In both datasets the values indicate nonlinearity between the epigenetic modulators and gene expression.
(EPS)

S5 Fig Model training and testing with H3K27Ac only emphasized the marker’s prominence in generalizing across the study’s GSC datasets. When the XGBR model is trained solely with the H3K27Ac marker data of GSC1 and tested with the other datasets, there is an observable increase in mean model performance for 10 of the 11 datasets. This visualization shows the results over 10 model runs, with 10 different seeds. The only dataset whose performance decreased is the study’s GSC2. This overall increase could be explained by a possible reduction in “noise” with the removal of the other epigenetic signals in training and the zeros that were proxies for those signals in testing.
(TIFF)

S6 Fig Spearman Correlation Coefficient experimental results. The SCC results visualized here support our PCC results where we found our cross-patient XGBoost-based model maintained the highest performance. The mean SCC values and their distributions over 10 experimental runs are shown for GSC1 as the training and GSC2 as the testing dataset.
(TIFF)

S7 Fig Spearman Correlation Coefficient metric comparative performance when model training with all features. When the cross-patient setup is trained with the study’s GSC1 dataset and all 4 epigenetic feature values, the SCC results fall below the corresponding PCC results for all the testing datasets.
(TIFF)

S8 Fig Spearman Correlation Coefficient metric comparative performance when model training and testing with the H3K27Ac feature only. When the cross-patient setup is trained and tested with H3K27Ac values only, we observe comparative SCC results to the prior testing.
(TIFF)

S9 Fig Epigenetic signal perturbation model performance comparison using XGBoost (GSC1 → GSC2). Each epigenetic signal was perturbed over 10 separate experiments. Shown are the mean PCC model results for 10 runs for each epigenetic marker (different random seeds) and error bars indicating the standard deviation. The hyperparameters used were identical to our other testing (see Table 1). The figures illustrate the order of model effect signal perturbation had from most to least: H3K27Ac, RNAPII, ATAC-seq, and CTCF.
(TIFF)

S10 Fig Spearman Correlation Coefficient results under feature perturbation. Our perturbation experiments produced SCC metric results alongside PCC results (S9 Fig). The results were compiled over the same 10 experimental runs, using our cross-patient XGBoost-based model and standard deviation indicated with error bars. Each epigenetic signal was perturbed separately when the model was trained with GSC1 and tested with GSC2. The model performance when H3K27Ac signals were perturbed was significantly decreased in comparison to the other signals.
(TIFF)

S11 Fig Expression category counts and Mean Squared Error values for GSC2 and Mack-GSC7. 10A) Visualizes the expression category counts of GSC2 where we see that the “low” category has a larger population than the “high” category. This gives us insight into the relatively low overall MSE that the model achieves when evaluating GSC2 (panel B). Mack-GSC7 has different proportions where the “low” category population is outnumbered by the “high” category (panel C). As a result, the overall MSE error is higher for this dataset overall and specifically in the gene categories we focus on.
(EPS)

S12 Fig Single-patient experimental results. The PCC results (panel A) when the model was trained and tested using splits of a single GSC dataset, were similar to those obtained from cross-patient setups. The mean PCC values for both datasets were higher than the SCC measurements (panel B).
(EPS)

S13 Fig Single-patient true/predicted values and feature importance. Panels A (GSC1) and B (GSC2) illustrate the testing dataset’s observed and predicted RNA-seq counts (after log2 transformation) per gene. The study found that although the models’ highest predicted values were lower than the dataset’s true values at the upper end of the range, the test set predictions trended towards the upper right indicative of model test set prediction accuracy across the range. The study also observed the fact that H3K27Ac was the most important feature for model prediction (panels C and D) which was in line with the cross-patient model behavior.
(EPS)

S14 Fig Single-patient gene expression category counts and mean squared error. Our GSC1 (panels S14A, S14B, S14C) and GSC2 (panels S14D, S14E, S14F) datasets both have higher numbers of “low” expression genes than “high” in both the training and testing sets. We observe low error rates for the “low” category and higher MSE for higher expression genes.
(EPS)

S15 Fig PCC cross-patient regression model over subsets of test data. The results are compiled as the mean PCC values, where testing is over 10 non-overlapping subsets of GSC2 (GSC1 → GSC2-subsets) and different random seeds of each model. The results align with the trends of Fig 4 and our cross-patient XGBoost-based regression model performed higher than all other architectures. The standard deviations of the result distributions shown here are higher than **Fig 4** while the distribution means are similar.
(TIFF)

S16 Fig SCC experimental model results when testing over subsets of GSC2 data. The mean SCC values and their distributions over 10 experimental runs are shown for GSC1 as the training and non-overlapping subsets of GSC2 as the testing dataset (GSC1 → GSC2-subsets). These results visualized here support our SCC result trends S6 Fig while all values are lower than the PCC counterparts. As it turns out, the MLP-based model’s mean is just above XGBR’s mean for this metric. As we observe in S15 Fig, the standard deviations of the distributions increase in comparison to S6 Fig with this method of testing.
(TIFF)

S17 Fig Feature importances from the XGBR cross-patient model mirror other results when testing over subsets of GSC2 data. The results visualized are nearly identical to those shown in **Fig 5**. H3K27Ac is identified as the most important feature for model prediction. These results are compiled by evaluating the model trained with GSC1 and evaluated using 10 non-overlapping subsets of GSC2 (GSC1 → GSC2-subsets) across a different random seed for each subset.
(TIFF)

S18 Fig Model training generalizes to other GSC datasets when evaluating over subsets of testing datasets. When we evaluate over subsets of the Mack et al [25]. derived datasets with the GSC1 trained model we observe the same generalization apparent in our other testing. Although the standard deviations of the results distributions are larger than S6 Fig, the means are within 0.031 PCC of each other.
(TIFF)

S19 Fig Spearman Correlation Coefficient metric illustrates model generalization after training with all features and evaluating over subsets of testing datasets. The SCC results visualization shows that the methodology generalizes across multiple GSC datasets. Although the values fall below their PCC counterparts, the means of each distribution are within 0.4 of each other. The standard deviations shown here are higher than when we evaluate the model using the entire testing dataset (S7 Fig).
(TIFF)

S20 Fig Model training and testing with the H3K27Ac marker alone successfully investigates the marker’s similarity among different GSCs. Testing the trained model across subsets of each GSC dataset produced the same trends in results observable when the entire testing is used together (S18 Fig). There is an increase in mean model performance in the Mack et al [25]. derived datasets when compared to **S5 Fig**. GSC2 is the only dataset whose mean PCC result decreased for the 10 model runs using different random seeds and data subsets.
(TIFF)

S21 Fig SCC results when training and testing (across subsets of testing data) with H3K27Ac only supports the other experiments. As with our other experiments using subsets of training data for 10 runs/random seeds, the standard deviations of the distributions are higher than those visualized in (S8 Fig) the trends are the same. Overall, there is only a 0.044 range over the values.
(TIFF)

S22 Fig Model evaluation across subsets of testing data is extensible to single-patient experimentation with similar results. We developed a variation of the GSC1 → GSC2-subsets methodology where both training and testing occurs within the same GSC dataset. This arrangement essentially becomes a within-patient cross-chromosomal setup with testing over subsets of hold-out data. In panel A there are the PCC results from the first parts of GSC1 and GSC2 as the training sets and parts 2–10 as separate test sets over different random seeds. As we note in the study’s other single-patient results (S12A Fig) the mean PCC is comparable to the various cross-patient results **(Figs 4, 6, and S5)**. Panel **B** visualizes the SCC results for the same set of experiments across subsets of the same dataset. The SCC values are below the PCC counterparts but support all the other values. This subset testing method produces standard deviations that are higher than the other testing while the overall model output remains aligned across all testing.
(EPS)

S23 Fig Feature importance in within-patient subset experiments find H3K27Ac to be the most important feature for gene expression prediction. H3K27Ac is the most important feature by a large margin in within-patient testing for both GSC1 **(A)** and GSC2 **(B)**.
(EPS)

S1 Table XGBoost Regression model hyperparameters. (XLSX)

S2 Table Multi-layered Perceptron hyperparameters. (XLSX)

S3 Table Branched Multi-layered Perceptron hyperparameters. (XLSX)

S4 Table Convolutional Neural Network hyperparameters. (XLSX)

S5 Table Recurrent Neural Network hyperparameters. (XLSX)

S6 Table Gradient Boosting Regression hyperparameters. (XLSX)

S7 Table Support Vector Regression hyperparameters. (XLSX)

S8 Table Multiple Linear Regression hyperparameters. (XLSX)

S9 Table Mean model script runtime measurements for experimental setup. (XLSX)

S10 Table Single-Patient XGBoost Regression model hyperparameters. (XLSX)

S11 Table Mean PCC increased for 10 of 11 test GSC datasets when H3K27Ac is the only feature. The percent change values for the Mack et al. datasets, each split into 10 non-overlapping sections, aligns with those from the other testing strategy (Table 1) [25]. GSC2 is the only dataset whose performance decreased when testing with H3K27Ac only. This supports the notion that the H3K27Ac signal alone is sufficient for strong gene expression prediction using this study’s experimental setup.
(XLSX)
