# Supplementary material for: Machine learning on multiple epigenetic features reveals H3K27Ac as a driver of gene expression prediction across patients with glioblastoma
Source: PLoS Comput Biol. 2025 Aug 7;21(8):e1012272. doi: 10.1371/journal.pcbi.1012272 (PMC12352877; doi:10.1371/journal.pcbi.1012272)
Supplement: S1 Text — Section S1. Epigenetic marker and RNA-sequencing information. Section S2. Flowcharts of data preparation and preprocessing process. Section S3. Supporting predictive models’ information [36–38]. Section S4. Correlation highlights the variation among the epigenetic features for each dataset. Section S5. Supporting model results. Section S6. Computational considerations. Section S7. Perturbation results support model feature importances. Section S8. Supporting gene expression count and model error analysis. Section S9. Single-patient experimental setup demonstrates comparable model prediction performance to cross-patient modeling and echoes the importance of H3K27Ac. Section S10. Model evaluation over subsets of data supports the trends uncovered in the results. (DOCX) [file pcbi.1012272.s001.docx]

**Supplementary Section**

**S1. Epigenetic marker and RNA-sequencing information.**

**GSC datasets:**

**Primary glioblastoma stem cell isolation and culture**. Primary human glioblastoma stem cells (GSCs) were isolated from Glioblastoma Multiforme (GBM) tumors using an established selection media-based protocol. GSCs were cultured in complete culture media: Neurobasal-A (Fisher Scientific, 10888022), B27 minus Vitamin-A (Fisher Scientific, 12587010), Glutamax (Fisher Scientific, 35050-061), Heparin (StemCell Tech, 07980), human HB-EGF, 100 ug (Peprotech, 100-47), human FGF,100 ug (Peprotech, 100-18B), and Antibiotic: Antimycotic (Anti-Anti) (Gemini Bio-products, 400-101).

**RNA-sequencing data.** GSCs were lysed using the Trizol reagent (Invitrogen). RNA was isolated from this lysate using the RNeasy mini kit (Qiagen). Next-generation sequencing was performed on these RNAs.

**RNA-pol II and H3K27Ac ChIP-sequencing data.** For each biological replicate, 5 million cells were pelleted and sent to Active Motif for chromatin immunoprecipitation, library preparation, and bioinformatic analysis. 75-nucleotide sequence reads were obtained using the Illumina NextSeq 500 system, resulting in over 30 million reads for each library, and aligned to the genome using the Burrows-Wheeler Alignment (BWA) algorithm. The 3’ end of aligned reads (tags) were extended in silico to 150-250 bp and the density of these extended fragments was quantified for the entire genome, which was divided into 32-nucleotide bins. The detection of peaks, genomic regions with significant local enrichment for tags, was performed using the MACS and SICER algorithms. Tag number across multiple libraries was normalized by being reduced to the number of tags in the smallest library through random sampling, in order to preserve site-specific and global differences between libraries.

**CTCF ChIP-seq.** For each biological replicate, 3 million cells were crosslinked and lysed using the truCHIP Chromatin Shearing Kit (Covaris). Isolated chromatin was sheared at 105 peak incident power, 2% duty factor, and 200 cycles per burst for 100 seconds in an S220 Focused-ultrasonicator (Covaris) to a length of 200-1200 bp. Shearing efficiency was validated using a Fragment Analyzer (Agilent). Immunoprecipitation for CTCF and ChIP-seq library preparation was performed using the ChIP-IT High Sensitivity Kit (Active Motif) and Next Gen DNA Library Kit (Active Motif), following the manufacturer’s instructions.

**ATAC-seq.** For each biological replicate, 50,000 cells were used to prepare ATAC-seq libraries in accordance with the protocol used in Ackermann et al. for alpha and beta cells, which was adapted with some modifications from the original protocol from Buenrostro et al. and the Omni-ATAC protocol. Samples with low viability (85%) were treated with DNase (Worthington) at a concentration of 200 U/mL for 30 minutes at 37° C. Double-sided bead purification to remove primer dimers and large 1,000 bp fragments was carried out using Agencourt AMPure XP beads (Beckman Coulter). Quality control was performed before sequencing using a Fragment Analyzer (Agilent) and the KAPA Library Quantification Kit (Roche). Libraries were analyzed by GENEWIZ using the Illumina HiSeq 2500 system to acquire 150 bp paired-end sequence reads. 200 million genomic reads per sample were obtained to detect open vs. closed chromatin regions.

**Mack et al. GSC data [25]:**

**H3K27Ac ChIP-sequencing data.** Our study makes use of data from the Stephen Mack Lab, Baylor College of Medicine. The 10 examples of H3K27Ac we use from their study are chosen randomly from the 44 sets of GSC data available. The data is available from the NCBI Gene Expression Omnibus under accession Series GSE119755 (<https://www.ncbi.nlm.nih.gov/geo/query/acc.cgi?acc=GSE119755>). The individual bedGraph files are available under separate accessions located at the following locations:

1) Mack-GSC7

GSM3382355

<https://www.ncbi.nlm.nih.gov/geo/query/acc.cgi?acc=GSM3382355>

2) Mack-GSC14

GSM3382285

<https://www.ncbi.nlm.nih.gov/geo/query/acc.cgi?acc=GSM3382285>

3) Mack-GSC18

GSM3382293

<https://www.ncbi.nlm.nih.gov/geo/query/acc.cgi?acc=GSM3382293>

4) Mack-GSC20

GSM3382299

<https://www.ncbi.nlm.nih.gov/geo/query/acc.cgi?acc=GSM3382299>

5) Mack-GSC25

GSM3382309

<https://www.ncbi.nlm.nih.gov/geo/query/acc.cgi?acc=GSM3382309>

6) Mack-GSC27

GSM3382313

<https://www.ncbi.nlm.nih.gov/geo/query/acc.cgi?acc=GSM3382313>

7) Mack-GSC35

GSM3382331

<https://www.ncbi.nlm.nih.gov/geo/query/acc.cgi?acc=GSM3382331>

8) Mack-GSC36

GSM3382333

<https://www.ncbi.nlm.nih.gov/geo/query/acc.cgi?acc=GSM3382333>

9) Mack-GSC38

GSM3382337

<https://www.ncbi.nlm.nih.gov/geo/query/acc.cgi?acc=GSM3382337>

10) Mack-GSC44

GSM3382349

https://www.ncbi.nlm.nih.gov/geo/query/acc.cgi?acc=GSM3382349

**RNA-sequencing data.** For gene expression counts, the data is adapted from accession Series GSE119834 (<https://www.ncbi.nlm.nih.gov/geo/query/acc.cgi?acc=GSE119834>). In this case, the raw counts data is available within a NCBI generated file (GSE119834_raw_counts_GRCh38.p13_NCBI.tsv.gz) located here: <https://www.ncbi.nlm.nih.gov/geo/download/?type=rnaseq_counts&acc=GSE119834&format=file&file=GSE119834_raw_counts_GRCh38.p13_NCBI.tsv.gz>. The individual accessions are as follows:

1) Mack-GSC7 GSM3384844

2) Mack-GSC14 GSM3384808

3) Mack-GSC18 GSM3384812

4) Mack-GSC20 GSM3384815

5) Mack-GSC25 GSM3384820

6) Mack-GSC27 GSM3384822

7) Mack-GSC35 GSM3384831

8) Mack-GSC36 GSM3384832

9) Mack-GSC38 GSM3384834

10) Mack-GSC44 GSM3384841

**S2. Flowcharts of data preparation and preprocessing process**

Our patient dataset preparation process involves a number of utilities which convert the feature reads housed in BAM files into counts for each of 50 bins for all 20,015 genes. As illustrated in S1 Fig, each of the four epigenetic signal data collections follow one preparation path while the expression data follows another. Ultimately, the two preparation paths converge to produce 2-dimensional, 1,000,750 x 5 files for model script input.

S2 Fig depicts our data preprocessing just prior to model input within the script. We illustrate that the epigenetic feature and target variable follow different paths for their respective preprocessing. The feature data leading to the dataset splitting process before standardization. Meanwhile, the target variable undergoes a log(2) transformation before the appropriate dataset splitting. The processes are coordinated to ensure that each gene’s information remains synchronized throughout.

To help facilitate our cross-patient prediction, the data preprocessing occurs in sequence for each patient datafile as the model script is run.

The datasets composed from Mack et al. information are preprocessed using a series of tools **(** **S3 Fig)** [25 ]. The process includes using the hg38 genome file (hg38.fa.gz), located at https://hgdownload.soe.ucsc.edu/goldenPath/hg38/bigZips/latest/, with samtools’ faidx to create an index. Since the Mack et al. H3K27Ac information is in the hg19 standard within bedGraph files, the CrossMap tool (https://crossmap.sourceforge.net) along with a hg19ToHg38.over.chain file is used to first realign the data to hg38 [25 ]. The aforementioned index and the realigned hg38 bedGraph is then used with bedtools’ bedToBam to create bam files, the same format that our study uses for the GSC1 and GSC2 epigenetic information before preprocessing. The bam files are then sorted and indexed to complete the H3K27Ac conversion process. The GSE119834_raw_counts_GRCh38.p13_NCBI.tsv.gz file containing raw RNA-seq counts mentioned in section (S1) is used because it includes data already converted to hg38 by NCBI. The GeneID numbers are translated into gene names using the NCBI provided annotation table named Human.GRCh38.p13.annot.tsv (https://www.ncbi.nlm.nih.gov/geo/download/?format=file&type=rnaseq_counts&file=Human.GRCh38.p13.annot.tsv.gz). Then this information is combined with the H3K27Ac measurements along with zeros used as proxies for CTCF, ATAC-seq, and RNAPII information. The data is then suitable to follow our study’s existing data preprocessing for model input.

**S3. Supporting predictive models’ information**

**XGBoost Regression (XGBR)**

An overview of the parameters we tune starts with $\eta$ (learning rate), which refers to the step size shrinkage of the feature weights at each iteration. "Max depth" refers to the maximum depth for each tree while "n estimators" determines the total number of trees in the model. $\gamma$ (min split loss) is the minimum reduction in loss required to split on a leaf node. A parameter that the algorithm uses to reduce overfitting is "subsample", the ratio of random samples taken from the training data before growing a new tree. The algorithm uses "colsample bytree" which is a ratio to determine the number of features to be randomly selected for each tree. Meanwhile, "min child weight" refers to the minimum number of samples (the weight) required to form a new tree node [[36,37](https://www.zotero.org/google-docs/?TBAaCf)].

**Multi-layered Perceptron (MLP)**

Our Multi-layered Perceptron model is one of our highest scoring configurations for PCC in our cross-patient experiments. Each gene’s epigenetic features are flattened and since batching is used, the dataset matrix changes from *b* x 50 x 4 to *b* x 200 where *b* represents the batch size. The model calculations then progress through a collection of three hidden (dense) layers which alternate with two dropout layers. The dropout layers provide regularization. The model’s 4th dense layer is configured with a single output (with linear activation) for RNA-seq value prediction.

This model’s hyperparameter tuning follows the same methodology as our other models and due to its architecture we tune each of the three dense layer sizes independently. Our tuning process results in an unconventional configuration where a downstream layer contains a higher number of nodes. We have a number of fine tuning experiments where the layer sizes change relative to each other and found that the existing setup performed well. Additionally, we opt to tune the dropout rate.

This model’s PCC and SCC results are consistently near the highest we observe with our data (GSC1→GSC2). Going further, it is the best performing of our deep learning based architectures in PCC. Because of that, we develop a multi-modal model (Branched MLP) based on this architecture for investigation.

**Branched Multi-layered Perceptron (Branched MLP)**

To investigate multi-modal computation’s effectiveness with our study’s gene expression prediction, we combine epigenetic features with genetic sequences for each gene in the dataset. The HG38 Human Reference Genome is used to create the sequence branch input. We acknowledge that the human reference genome may not be an informative source to partner with our glioblastoma epigenetic measurements since they are each derived from different cell types.

The preparation process of the gene sequence data, outside of the script, uses the same BED location files we used in our epigenetic data preparation process. The goal being to ensure that the location for each gene sequence exactly matches the epigenetic markers. For the sequences we collect 100 base pairs for each of the 50 bins. The sequences are converted to numerical numerical representations as follows:

A = 1, T = 2, C = 3, G = 4, N = 5

These steps lead to numpy files that we use as additional input into the model script. The 3-dimensionally structured sequence data’s index position is an exact match to its corresponding epigenetic measurement position.

To take into account the computational complexity of this model the number of bins is reduced to 21. Since TSS is located at bin 25 the sequence input ranges from bins 15 to 35 producing an intermediate matrix shape of 20,015 x 21 x 100. Finally, our script one-hot encodes the numerical representations in the following way:

1 = [1, 0, 0, 0, 0], 2 = [0, 1, 0, 0, 0], 3 = [0, 0, 1, 0, 0], 4 = [0, 0, 0, 1, 0], 5 = [0, 0, 0, 0, 1]

The model uses MSE for loss calculations and Adam optimization. We batch the input into the model’s respective “epigenetic” and “sequence” branches. Both branches have 3 dense layers with dropout layers in place for regularization. The output of those two branches is concatenated and then goes into 4 dense layers. The first 3 layer sizes are tuned along with those in the aforementioned branches. The final dense layer is a single output for RNA-seq prediction.

This model achieved PCC and SCC scores that are generally improved over the Multiple Linear Regression and Support Vector Regression but overall does not perform above the others. We also note that the standard deviation of its scores is higher than the others which may indicate that its predictions are less stable than our other models.

Although we find other models to be more predictive in our study, multi-modal approaches like this one are interesting for future investigation.

**Convolutional Neural Network (CNN)**

We apply various configurations of Tensorflow/Keras based Convolutional Neural Network (CNN) model architectures to our cross-patient prediction task as well. As with our MLP model we use MSE for loss calculations and Adam optimization. The CNNs also use Dropout layers for regularization.

Our implementations include 1, 2, and 3 Conv1D layer versions with MaxPooling1D pooling layers. After the convolutional modules there are a series of dense layers which lead to final prediction output. Our final versions use ReLU activation although we ran various experiments using LeakyReLU. Ultimately, the 1 layer results are reported in the study because it received the most extensive tuning, training and testing within this group of models.

The nature of CNN layers and batching when fitting the model, allows us to keep the datasets in their 3-dimensional structure for input. The CNN layer output is only flattened just prior to the model’s dense layers.

**Recurrent Neural Network (RNN)**

The sequential nature of the epigenetic features make Recurrent Neural Networks (RNNs) a suitable model choice for their capability to capture spatial relationships within the sequence. We conduct experiments with various RNNs and their extensions, and determine that AttentiveChrome, based on Gated Recurrent Units (GRUs) instead of Long Short-Term Memory (LSTM) [[38](https://www.zotero.org/google-docs/?SSGHqB)], achieves the best performance on the dataset.

The AttentiveChrome architecture incorporates two levels of RNN and an attention mechanism. The first level consists of four GRUs, each processing information about one epigenetic feature. The input to these GRUs is treated as a time series, with each element in the sequence (i.e., each bin value for each epigenetic feature of a single gene) representing a time step (*x_t_*), with *t* ranging from 1 to *T* (*T*=50, as there are 50 bins). GRUs, as all other RNNs, maintain an internal state called the hidden state (*h*), which captures the historical information of the input sequence up to the current step. This hidden state acts as a form of memory, enabling GRUs to retain information about the observed data in the sequence and capture temporal (in terms of epigenetic features, spatial) dependencies. At each time step, the GRUs update their hidden state (*h*_t_) by considering the current input (*x*_t_) and the hidden state from the previous step (*h*_t−1_). GRUs employ two activation functions, functioning as an update gate and a reset gate, respectively, to selectively retain and erase information in the hidden state, mitigating the issue of vanishing gradients.

To enhance the GRUs' ability to capture long-term dependencies and focus on relevant segments of the input sequence, a soft attention layer is applied to the hidden states of all time steps (*h*_1_*h*_2_…*h*_T_) from each of the four GRUs. The attention mechanism assigned weights to the hidden states, reflecting their relative importance, and thus add complexity and interpretability. The results are then concatenated and subsequently passed through a new GRU layer (with 4 time steps corresponding to the 4 epigenetic features), an additional attention layer, and a dense layer to generate the final predicted gene expression value. TensorFlow Keras is employed for the implementation of this model.

**Gradient Boosting Regression (GBR)**

The Gradient Boosting Regression (GBR) model performance after tuning is comparable to our deep learning models in the cross-patient experimental setup. This is an early indication of the potential of tree based algorithms for prediction with our epigenetic patient data.

The model implementation lacks graphical processing unit (GPU) acceleration and although the run times are competitive with the other models, this leads us to consider the application of similar algorithms with GPU acceleration available.

**Support Vector Regression (SVR)**

To implement a Support Vector Machine based model we utilize the scikit-learn library’s version. The datasets are reshaped, for model input using the identical method to our other traditional machine learning algorithm setups.

Our tuning is centered on C and $\epsilon$ hyperparameters where we find 0.01 to be the optimal valve for both.

Across all of our testing, the SVR outperforms linear regression but does not reach the levels of PCC achieved by other models.

**Multiple Linear Regression (MLR)**

Our linear regression model is implemented using the statsmodels Application Programing Interface (API) using the Ordinary Least Squares (OLS) version of the algorithm. Since this is a traditional machine learning algorithm, our 3-dimensional datasets are reshaped into 2 dimensions before model input.

We tune both the alpha (penalty weight) and L1_wt (the portion of the penalty applied to the L1 term). Additionally, elastic net regularization is used.

**S4. Correlation highlights the variation among the epigenetic features for each dataset**

To see if the application of machine and deep learning is appropriate to model the relationship between these epigenetic sequencing data and RNA-seq, we performed correlation analysis between gene expression and each epigenetic modulator (ATAC-seq, H3K27Ac ChIP-seq, CTCF ChIP-seq, and RNAPII ChIP-seq). To accomplish this, our patient datasets are each prepared in a similar fashion to what occurs in our models with some notable differences. The RNA-seq values are not log(2) transformed and the epigenetic feature data are not standardized. We instead calculate the sum of the bin counts for each gene’s 4 features where each row represents a gene’s bin position.

Our results indicate a positive correlation between gene transcription and each epigenetic modulator, with PCC values of 0.366, 0.111, 0.208, and 0.333 (for H3K27Ac, CTCF, ATAC-seq, and RNAPII, respectively) in GSC1, and PCC values of 0.412, 0.162, 0.221, and 0.359 (for the same order of modulators) in GSC2 (S4A and S4B Fig). These findings provide a promising starting point for exploring the potential of ML/DL models in this domain, based on the non-linearity between each modulator and RNA-seq which more complex machine and deep learning models can investigate.

**S5. Supporting model results**

Our model results when using all four epigenetic features for RNA-seq prediction and the observation that H3K27Ac is the most important among them is encouraging a deeper investigation of the marker. In Section 4.4 we outline the results we obtain when training and testing with H3K27Ac only **(** **S5 Fig)**. We observe that when we compare them to the results of Section 4.3 **(Fig 6)** the model’s PCC for the majority of the datasets (the exception is GSC2) increases. We detail the general trend of PCC increase and the decrease of GSC2 in Table 1 of Section 4.4.

All of our experiments produce Spearman Correlation Coefficient (SCC) results along with PCC. We designate it as our secondary evaluation metric and during our analysis, we note that while many models’ values are comparatively lower than the corresponding PCC findings, the trends in how the models perform relative to each other remain similar **(** **S5 Fig)**. Our highest scoring model under PCC, XGBR, has the highest SCC results as well. Additionally, we perform the same analysis, for cross-patient testing when training with GSC1’s entire featureset **(** **S7 Fig)** and H3K27Ac only **(** **S8 Fig)**. It should be noted here that the hyperparameter combinations are optimized for PCC and not SCC. With that in mind, it’s interesting that the results we see support our PCC observations.

**S6. Computational considerations**

Script runtime is also taken into consideration as a measure of computational impact. The time it takes for the experiments to be complete can provide some information towards its environmental impact and the resource availability. While we acknowledge the particular factors we consider within our environment, we also can not assume that future work in this space would not be influenced in some way by computational impact.

Our computational times are not intended to be taken as absolute. These run times are measured according to the elapsed time for the python script (containing the model and associated functions) to run, as calculated by the computational platform. For example, our deep learning scripts include functions for tasks such as gene level error recording, and visualization to name a few. Additionally, since the computational platform is a shared resource, run times can be influenced by the load of other research tasks at a given time of day. Additionally, some of the model architectures use Graphical Processing Unit (GPU) acceleration which will impact many of the calculations and therefore reduce the runtime.

The model script runtime found the MLR to complete the fastest followed by XGBR’s script completion time. The other models, with the exception of one, are comparatively close to both the MLR and XGBR times. The Branched MLP performs behind the others in this regard. The Branched MLP’s time is affected not only by the fact that the 3-dimensional matrix of gene sequences are formed when the model is run but also by the one-hot encoding of that branch input. Together with the computational complexity, the runtime of the Branched MLP is over five times the MLR. We note that the XGBR model has the dual advantage of leading in metric performance while the script also completes the predictions faster than the majority of the others.

**S7. Perturbation results support model feature importances**

To evaluate the contribution of each epigenetic marker to predicting gene expression, we conduct perturbation experiments on each marker and observe the resulting performance metrics (PCC). The model is trained with GSC1 and evaluated on GSC2 (GSC1→GSC2) where for each feature separately, all the values are changed to 0 over 10 runs/random seeds of the model. The most striking change occurs when we perturb the H3K27Ac signals. We observe a decline in performance of 0.583851 (70.667%) in PCC. Additionally, we note declines of 0.036298 (4.393%) for RNAPII, 0.004939 (0.597%) for ATAC-seq, and 0.00419 (0.507%) for CTCF perturbations **(** **S9 Fig)**. This suggests that the predictive dependence of gene transcription is on all epigenetic markers (histone modifications, RNAPII binding, broad chromatin accessibility, and chromatin looping), with a greater weight on H3K27Ac signals followed by RNAPII, ATAC-seq, and then CTCF.

Comparing these PCC values to the correlation values between RNA-seq and each epigenetic marker, we can say that our cross-patient prediction analysis captured a trend that is not captured by simple correlation analysis **(** **S4A**  **and S4B Fig)**. Additionally, the importance of H3K27Ac signals is further supported by the SCC metric **(** **S10 Fig)** and the primary model’s feature importance output **(Fig 5)**. Just as with PCC, perturbing H3K27Ac produces the greatest SCC metric change. Interestingly, the perturbation of CTCF causes an increase in the metric by 0.000354. One explanation for this may be the fact that the models are not optimized for SCC. Therefore, although other features are impactful, albeit to lesser degrees, H3K27Ac perturbation produces the greatest performance change positively or negatively.

**S8. Supporting gene expression count and model error analysis**

In Section 4.5 we analyze the mean H3K27Ac marker signal in 3 settings: “high” expression genes, “low” expression genes and all the genes in each of our datasets. Our training dataset, GSC1, contains 68 “high” expression genes, along with 9880 genes in our “low” expression category.

To further investigate this we choose to examine GSC2 as our study’s original dataset and one from the Mack et al. based datasets we compiled, Mack-GSC7 [25 ]. GSC2’s gene expression category distributions **(** **S11A Fig)** closely match GSC1’s. As a result there are both strong PCC results and overall low error rates **(** **S11B Fig)**. It’s logical that the error rate is higher for GSC2’s “high” expression genes since the model is exposed to a comparatively low number of “high” genes during training. When we examine Mack-GSC7 we note that the overall MSE is higher than GSC2’s **(** **S11C**  **and S11D Fig)**. The fact that the model is able to generalize to data outside of the training distribution such as Mack-GSC7 may be explained by its ability for low error in the “low” category by virtue of its training.

**S9. Single-patient experimental setup demonstrates comparable model prediction performance to cross-patient modeling and echoes the importance of H3K27Ac.**

To fully evaluate the study’s original datasets, both GSC1 and GSC2, the XGBoost architecture is used in a single dataset (within-patient cross-chromosomal) experimental setup. Both datasets are made up of 20,015 sets of gene observations where each one is composed of 50 measurements (bins) for each of the 4 epigenetic features. The total samples per dataset amount to 1,000,750 for each marker. After employing a 70/30 data split with 14,010 (700,500 samples) genes in the training and 6,005 (300,250 samples) in the testing set, the data is flattened for input. The experiments are run 10 times using 10 different random seeds using the same hyperparameters employed in the cross-patient version (S10 Table).

The PCC results reinforced the cross-patient values where GSC1 is 0.808759 ± 0.000459 while GSC2 achieves 0.826806 ± 0.0005920 **(** **S12A Fig)**. The SCC results **(** **S12B Fig)** are similar for GSC1 (0.802057 ± 0.000588) and GSC2 (0.812661 ± 0.000650). H3K27Ac is the most important feature for both datasets (S13C and S13D Fig). Finally, true/predicted RNA-seq plots **(** **S13A and S13B Fig)** closely resemble the GSC2 plots of the cross-patient experiments **(Fig 7A)**.

The results of these experiments support our cross-patient testing where we find that in our study’s datasets the “low” gene expression group outnumbers “high” expression in both the single-patient GSC1 **(** **S14A and S14B Fig)** and GSC2 **(S14D and S14E Fig)**. GSC1 contains 6980 “low” expression genes in training, 2900 in testing. Meanwhile, it has 52 and 16 in the training and testing “high” category. Conversely, GSC2 has 6706 and 2791 in the “low” training and testing groups and 51 versus 18 in the “high” categories. In S14C and S14F Fig we provide examples of the MSE analysis of these experiments. In both cases the overall MSE of these (2.800390 and 2.586611) is similar to the error achieved from the GSC2 dataset in cross-patient testing (2.691721) shown in S11B Fig.

**S10. Model evaluation over subsets of data supports the trends uncovered in the study’s results.**

To investigate an alternative experimental setup where the trained model is evaluated using subsets of the testing datasets, the datasets are separated into different files as outlined in 3.5. As a result, the means and standard deviations (SD) are calculated over these subsets of hold-out genes as opposed to the entire hold-out set multiple times **(Figs 4, 5, 6, and Table 1)**. The study is able to adapt this approach into both cross-patient (ie, GSC1→GSC2-subsets) and single-patient scenarios.

In cross-patient (GSC1→GSC2-subsets) evaluation, the XGBR model has the highest mean PCC value out of the suite of models in the study **(** **S15 Fig)**. The mean for this model - 0.824419$\pm$0.022086 - is 0.00596 above the next closest model (GBR, 0.818456$\pm$0.022061). Meanwhile, it is 0.147661 PCC higher than the least performant algorithm (MLR, 0.676758$\pm$0.030888). These mean values are in line with the original study findings **(Fig 4)**. Specifically, the mean across all of the models using the original strategy is 0.78041 while the subset strategy produces a mean of 0.78203.

Interestingly, a product of this approach is an increase in nearly all the result distributions’ standard deviations. The mean SD across all models in the original setup is 0.003857. Conversely, the subsets calculations yield a mean SD of 0.023850. The only SD to decrease is the Branched MLP which went from SD 0.021115 to 0.019820.

The SCC results under these experiments very closely resemble the trends observed in the original SCC testing. **S16 Fig** visualizes the distributions whose increase in SD is evident compared to **S6 Fig**. Although the individual means are lower than their PCC counterparts above and XGBR is not the highest performer with this strategy and metric, it consistently produces strong results when compared overall.

When we continue this analysis by examining the XGBR feature importance, we observe H3K27Ac to be the model’s most important epigenetic feature. In the original testing strategy, the feature has a mean of 0.750977 **(4.2)**. Testing over subsets produces a mean value of 0.753481. In fact, the order of feature importance noted in that section is consistent here with RNAPII, ATAC-seq, and CTCF following in that order **(** **S17 Fig)**.

When we extend this testing strategy to the Mack et al derived datasets, our discussion of CIPHER’s ability to generalize using different datasets applies [25 ]. We find that under both the PCC **(** **S18 Fig)** and SCC **(** **S19 Fig)** metrics, the mean scores are within 0.30403 and 0.035987 of each other respectively. Meanwhile, the average SD for PCC is 0.017902 and for SCC is 0.019622. The means are within the standard deviations of each other. Therefore, we can infer that the different GSC datasets perform similarly to each other within a framework related to CIPHER but using subsets of testing data.

Additionally, just as with the CIPHER framework, we observe that model training while testing with subsets using H3K27Ac alone can be effective in gene expression prediction. PCC results **(** **S20 Fig)** display a similar trend to previous testing where there is an increase in the metric when focused on the one feature. The percent change is again an increase by roughly 2% **(** **S11 Table)**.

When we divide the testing datasets into smaller subsets as individual files, it allows us to apply the cross-patient framework in a single-patient scenario. To maintain consistency in these experiments, the XGBR model is trained with “part 1” of the datasets and evaluated with “parts” 2 to 10, with a different random seed for each. Therefore, we achieve a similar experimental setup that satisfies both within-patient and across-subset testing criteria. As it turns out, our notion of generalizability is supported here as well, and with a similar trend. Specifically, both PCC **(** **S22A Fig)** and SCC **(** **S22B Fig)** have similar mean values for the distributions as our original testing strategy **(** **S12A and S12B Fig),** even though the standard deviations are higher here. Additionally, H3K27Ac is again the dominant epigenetic feature for prediction by significant margins for both GSC1 **(** **S23A Fig)** and GSC2 **(** **S23B Fig)**. All of these within-patient results support the cross-patient testing, where the strategy is to evaluate the trained model with a single testing dataset over multiple random seeds or over subsets of said datasets.
