# Supplementary figures and images for: Machine learning on multiple epigenetic features reveals H3K27Ac as a driver of gene expression prediction across patients with glioblastoma
Source: PLoS Comput Biol. 2025 Aug 7;21(8):e1012272. doi: 10.1371/journal.pcbi.1012272 (PMC12352877; doi:10.1371/journal.pcbi.1012272)

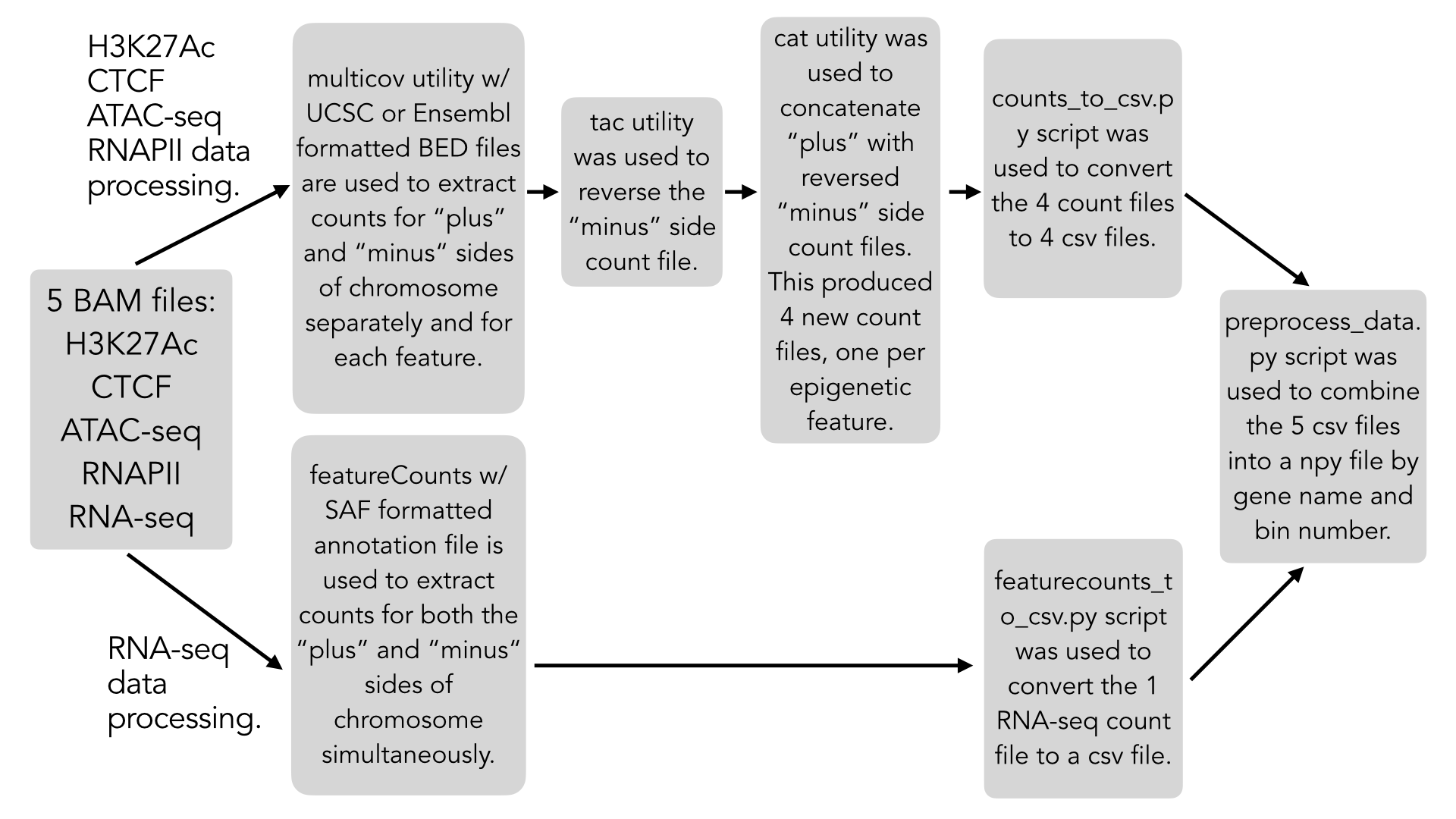

Supplement: S1 Fig — The process that extracts the observed epigenetic marker values from BAM format files uses a series of utilities which combine the separate data into one file. The process aligns the 50 bin values of the gene’s epigenetic features with a single extracted RNA-seq value for the gene to maintain consistent formatting. (TIFF) [file pcbi.1012272.s002.tiff]

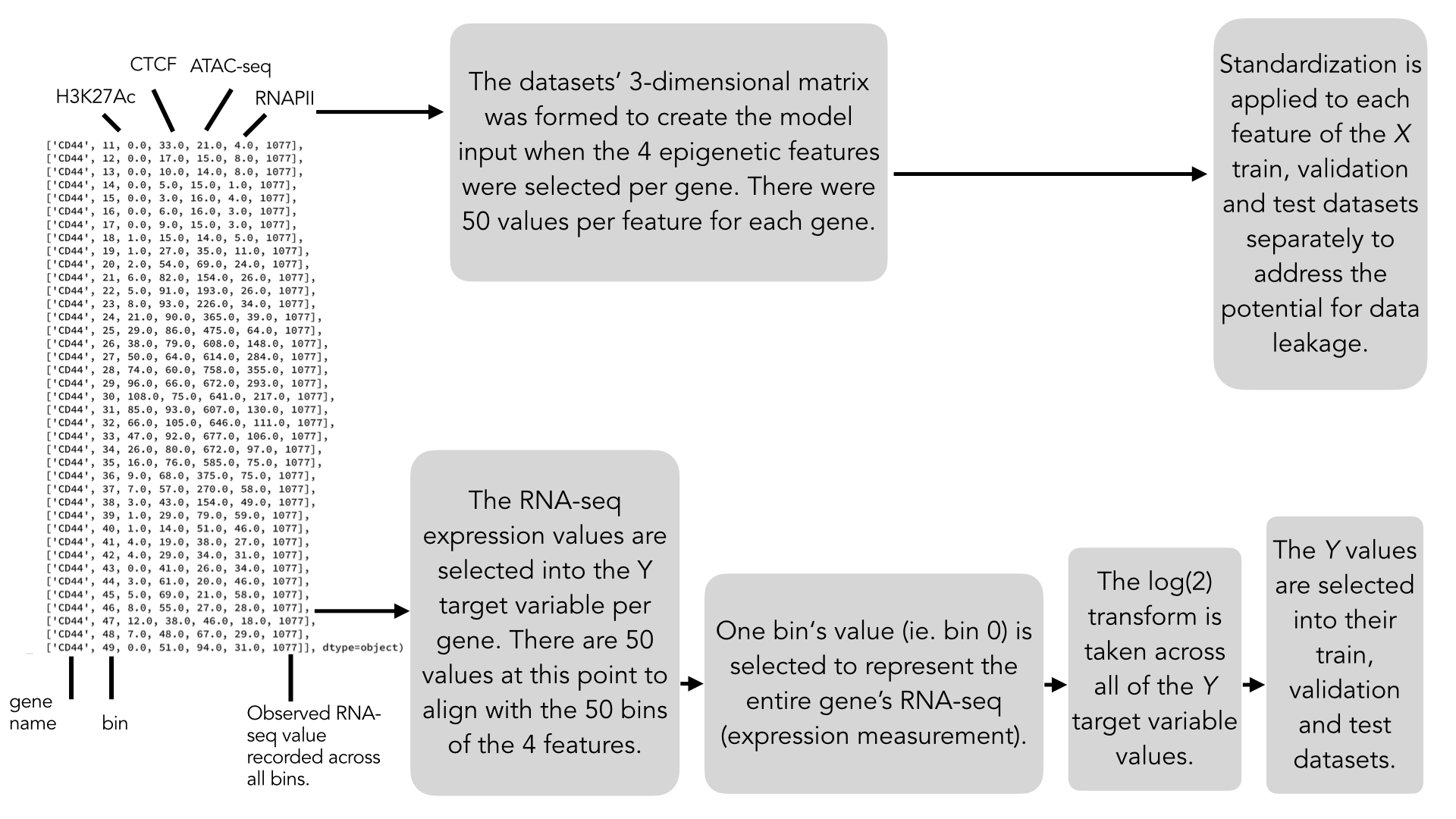

Supplement: S2 Fig — The epigenetic feature values per gene are extracted, formed into matrices, and standardized for model input. Each gene’s observed RNA-seq value is extracted and transformed creating the target variable for the model. (TIFF) [file pcbi.1012272.s003.tiff]

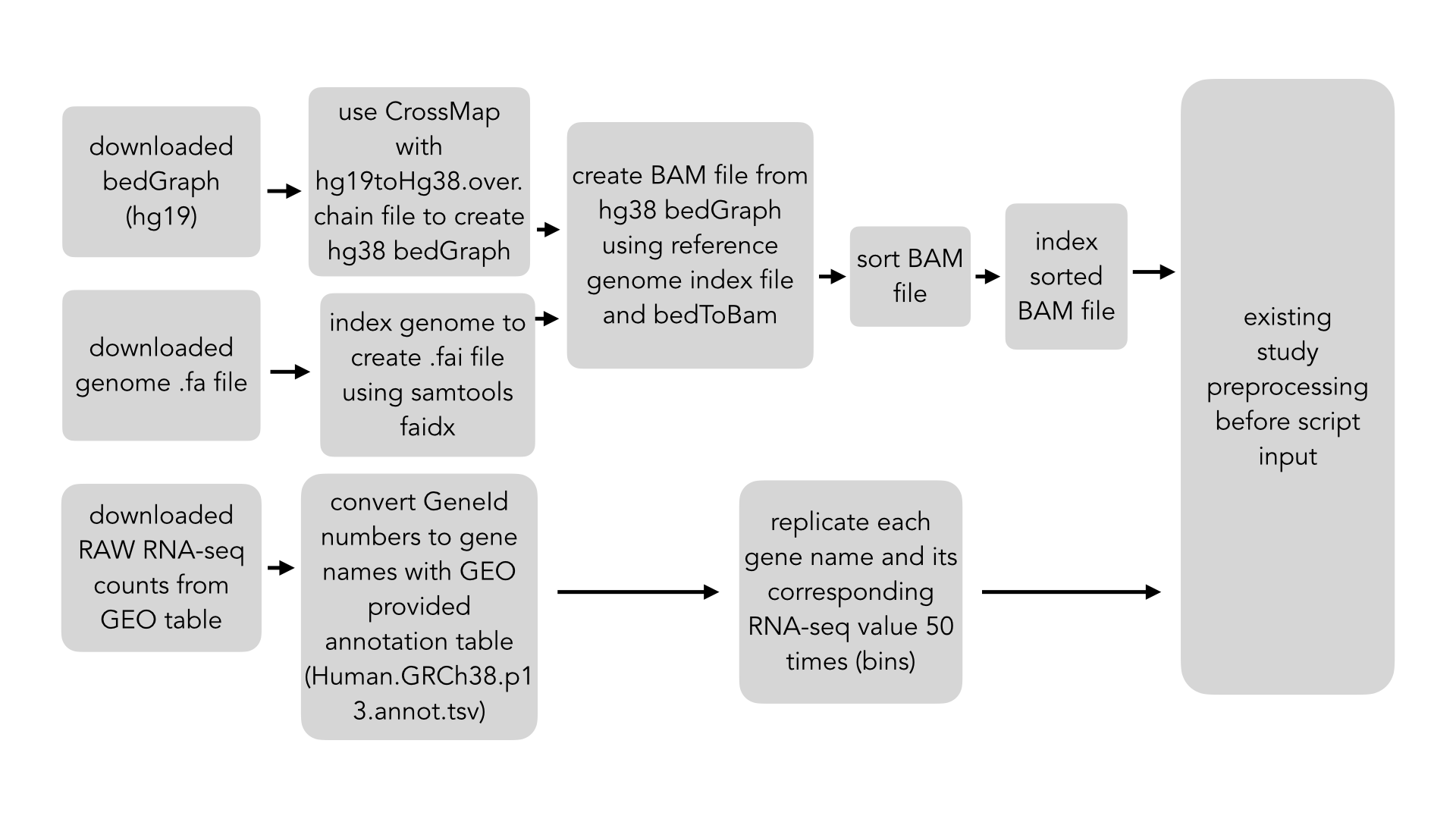

Supplement: S3 Fig — We use this process to convert and adapt the downloaded H3K27Ac and RNA-seq information to align with our study’s existing preprocessing. (TIFF) [file pcbi.1012272.s004.tiff]

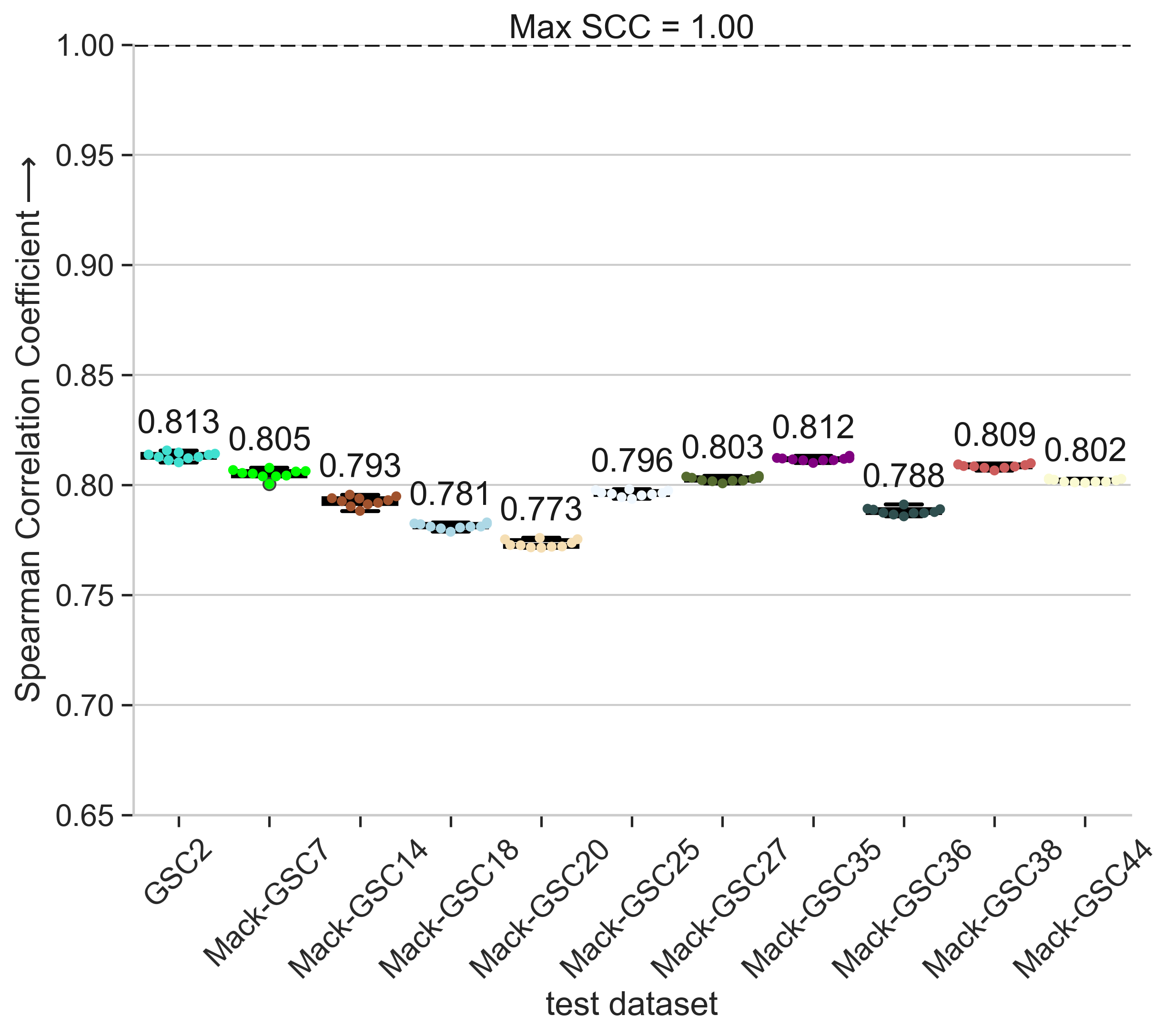

Supplement: S7 Fig — When the cross-patient setup is trained with the study’s GSC1 dataset and all 4 epigenetic feature values, the SCC results fall below the corresponding PCC results for all the testing datasets. (TIFF) [file pcbi.1012272.s008.tiff]

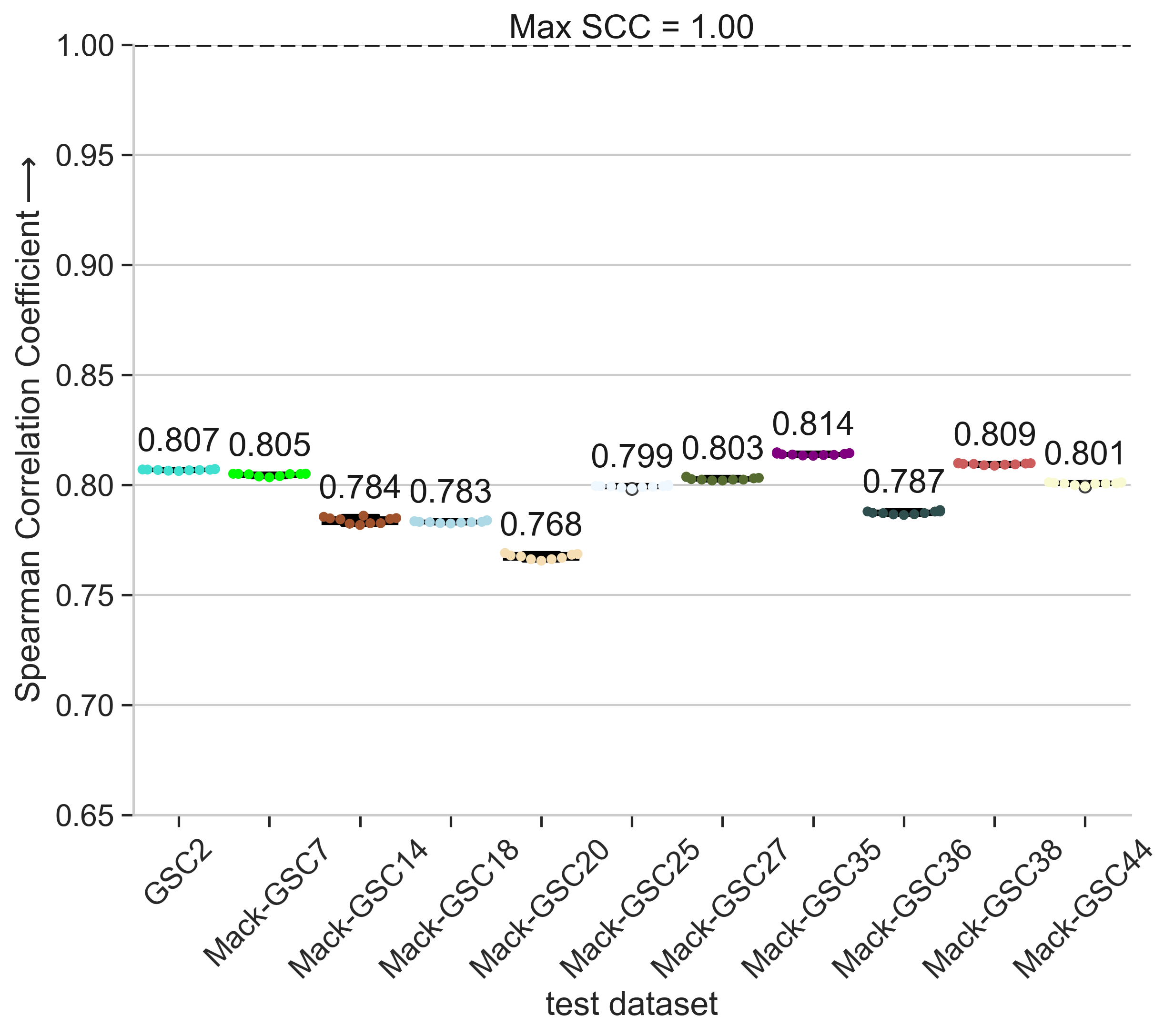

Supplement: S8 Fig — When the cross-patient setup is trained and tested with H3K27Ac values only, we observe comparative SCC results to the prior testing. (TIFF) [file pcbi.1012272.s009.tiff]

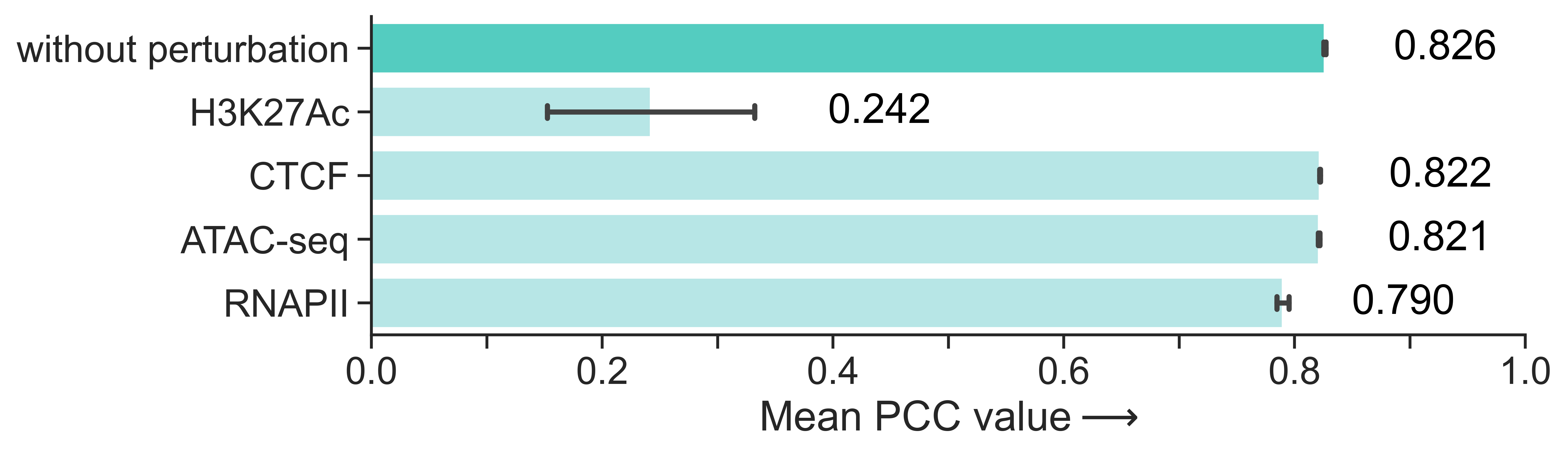

Supplement: S9 Fig — Each epigenetic signal was perturbed over 10 separate experiments. Shown are the mean PCC model results for 10 runs for each epigenetic marker (different random seeds) and error bars indicating the standard deviation. The hyperparameters used were identical to our other testing (see Table 1). The figures illustrate the order of model effect signal perturbation had from most to least: H3K27Ac, RNAPII, ATAC-seq, and CTCF. (TIFF) [file pcbi.1012272.s010.tiff]

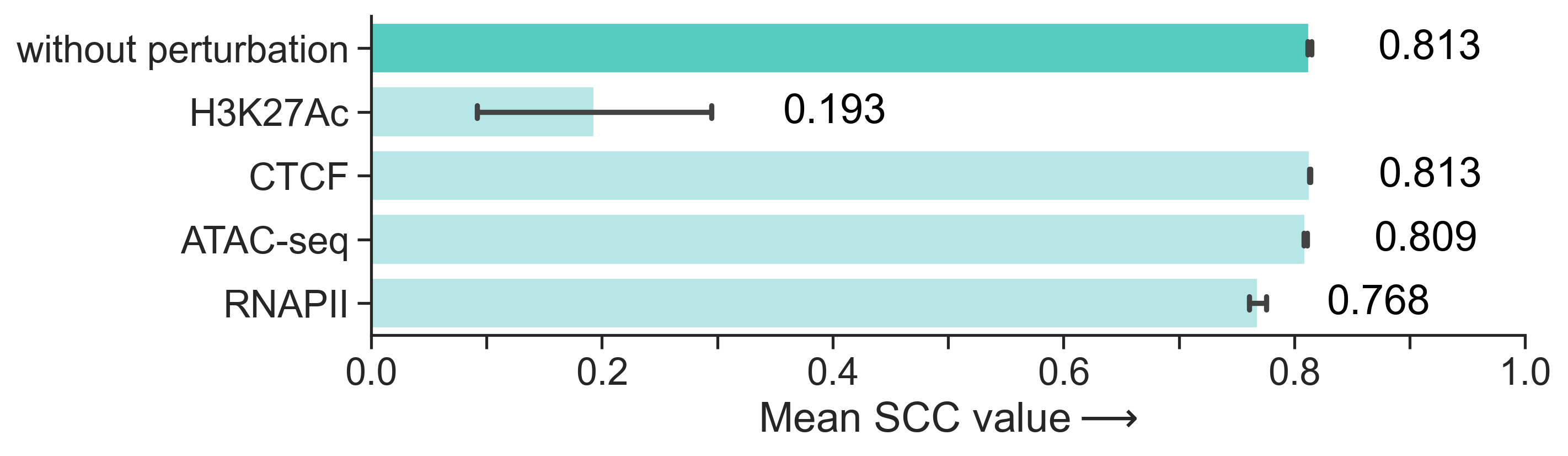

Supplement: S10 Fig — Our perturbation experiments produced SCC metric results alongside PCC results (S9 Fig). The results were compiled over the same 10 experimental runs, using our cross-patient XGBoost-based model and standard deviation indicated with error bars. Each epigenetic signal was perturbed separately when the model was trained with GSC1 and tested with GSC2. The model performance when H3K27Ac signals were perturbed was significantly decreased in comparison to the other signals. (TIFF) [file pcbi.1012272.s011.tiff]

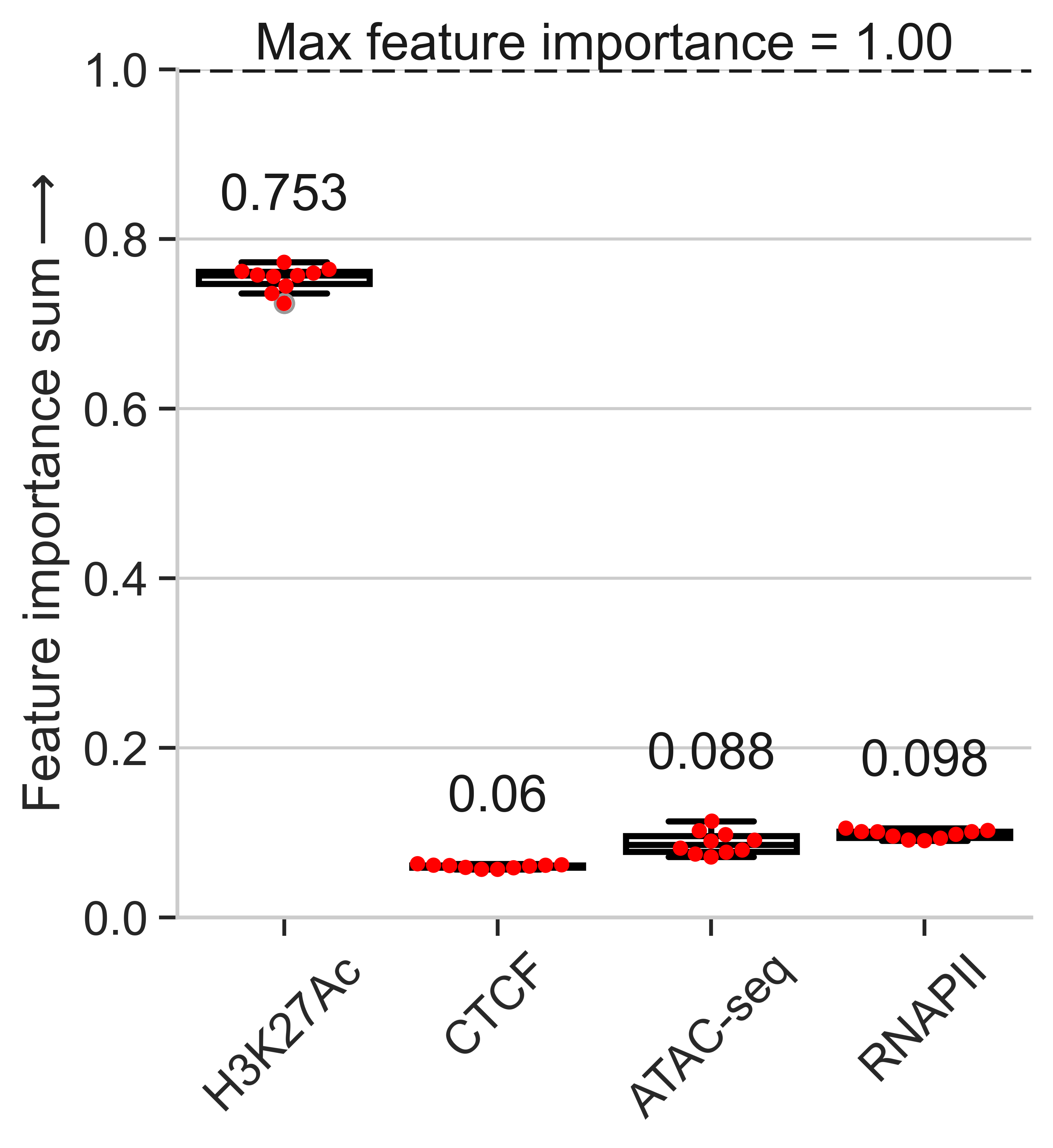

Supplement: S17 Fig — The results visualized are nearly identical to those shown in Fig 5. H3K27Ac is identified as the most important feature for model prediction. These results are compiled by evaluating the model trained with GSC1 and evaluated using 10 non-overlapping subsets of GSC2 (GSC1 → GSC2-subsets) across a different random seed for each subset. (TIFF) [file pcbi.1012272.s018.tiff]
